# Alternative Transmission Patterns in Independently Acquired Nutritional Cosymbionts of Dictyopharidae Planthoppers

**DOI:** 10.1128/mBio.01228-21

**Published:** 2021-08-31

**Authors:** Anna Michalik, Diego Castillo Franco, Michał Kobiałka, Teresa Szklarzewicz, Adam Stroiński, Piotr Łukasik

**Affiliations:** a Department of Developmental Biology and Morphology of Invertebrates, Institute of Zoology and Biomedical Research, Faculty of Biology, Jagiellonian University, Krakow, Poland; b Institute of Environmental Sciences, Faculty of Biology, Jagiellonian University, Krakow, Poland; c Museum and Institute of Zoology, Polish Academy of Sciences, Warsaw, Poland; Univ. California Merced; University of Hawaii at Manoa

**Keywords:** planthoppers, nutritional symbiosis, transovarial transmission, genomes

## Abstract

Sap-sucking hemipterans host specialized, heritable microorganisms that supplement their diet with essential nutrients. These microbes show unusual features that provide a unique perspective on the coevolution of host-symbiont systems but are still poorly understood. Here, we combine microscopy with high-throughput sequencing to revisit 80-year-old reports on the diversity of symbiont transmission modes in a broadly distributed planthopper family, Dictyopharidae. We show that in seven species examined, the ancestral nutritional symbionts *Sulcia* and *Vidania* producing essential amino acids are complemented by co-primary symbionts, either *Arsenophonus* or *Sodalis*, acquired several times independently by different host lineages and contributing to the biosynthesis of B vitamins. These symbionts reside within separate bacteriomes within the abdominal cavity, although in females *Vidania* also occupies bacteriocytes in the rectal organ. Notably, the symbionts are transovarially transmitted from mothers to offspring in two alternative ways. In most examined species, all nutritional symbionts simultaneously infect the posterior end of the full-grown oocytes and next gather in their perivitelline space. In contrast, in other species, *Sodalis* colonizes the cytoplasm of the anterior pole of young oocytes, forming a cluster separate from the “symbiont ball” formed by late-invading *Sulcia* and *Vidania*. Our results show how newly arriving microbes may utilize different strategies to establish long-term heritable symbiosis.

## INTRODUCTION

Mutualistic relationships with heritable bacterial and/or fungal microorganisms have played crucial roles in the biology of multiple groups of insects, contributing significantly to their evolutionary and ecological success (1–3). The growing awareness of the diversity and importance of insect symbioses, in addition to the rapid development in sequencing-based techniques, has led to an increased interest in these associations. However, outside a few model species and some reasonably well-sampled clades, our knowledge of the diversity, evolution, and biological characteristics of the microbial symbionts and the microbial roles in the evolution of insect diversity remains limited (4, 5). Among insects, sap-sucking hemipterans are obligately dependent on heritable nutritional microbes that supplement their unbalanced diet with essential amino acids, vitamins, and cofactors (4, 6–8). Multiple symbiont combinations have been described from Auchenorrhyncha, a suborder comprising infraorders Fulgoromorpha (planthoppers) and Cicadomorpha (cicadas, spittlebugs, treehoppers, and leafhoppers) (4, 9). Their common ancestor that lived about 300 million years ago (MYA) is thought to have been colonized by two microbes, a *Bacteroidetes* member currently known as “*Candidatus* Sulcia muelleri” (further referred to as *Sulcia*) and a betaproteobacterium, variably known as “*Ca.* Nasuia deltocephalinicola,” “*Ca.* Zinderia insecticola,” or “*Ca.* Vidania fulgoroideae” (further referred to as *Nasuia*, *Zinderia*, and *Vidania*, respectively, or as beta-symbionts collectively) (9–12). These maternally transmitting and nutrient-providing symbionts have become obligate components of host biology. However, in many host clades, one or both became complemented or replaced by other microbes. In these multipartite symbioses, microbes share the responsibility for essential nutrient biosynthesis. For example, in known Cicadomorpha, *Sulcia* encodes pathways for producing 7 or 8 essential amino acids, whereas the remaining 3 or 2 amino acids are provided by its symbiotic partner (8, 9, 12). The situation can get more complicated when one of the ancient microbes gets replaced by another or when the host is colonized by more than two symbionts, and the nutritional functions become subdivided among a greater number of partners. This has occurred repeatedly in different lineages of Auchenorrhyncha, where additional symbionts have either taken over the beta-symbiont’s role or contribute to vitamin biosynthesis (4).

These intimate and intricate metabolic interdependencies between insects and associated microorganisms indicate a vital role of nutritional symbionts in host biology and the need to ensure reliable transmission of these essential partners across generations. Microbes can be transmitted from mothers to offspring (vertically) or through the environment (horizontally), and within these transmission modes, a variety of transmission routes exists (13). In sap-feeding hemipteran insects, transovarial transmission through female germ cells, at a certain stage of their development, predominates (14–16). In some insects, symbionts infect undifferentiated germ cells, or young, previtellogenic oocytes (16). However, in most hemipteran taxa, including all Auchenorrhyncha studied to date, they invade ovarioles containing older (vitellogenic or choriogenic) oocytes. The complementation or replacement of ancient, coadapted heritable nutritional symbionts by newly arriving microbes, while likely beneficial to the hosts because of their greater metabolic capacity and efficiency (17, 18), creates apparent challenges for their transmission. Host lineages that acquired new symbionts have to adopt existing mechanisms or develop new traits and mechanisms for their effective vertical transmission, which is crucial for the fixation of new symbiosis (19), being also a matter of life or death to the newly established symbionts. The evolution of the symbiont transmission and the symbiont replacements are inseparably linked, and we need to study one to understand the other.

While summarizing decades of microscopy-based research on Auchenorrhyncha symbioses, Buchner (14) famously wrote about “the veritable fairyland of insect symbiosis,” apparently referring to the diversity of microbes in different host clades, as well as their transmission mechanisms. However, he lacked tools to fully characterize the evolution of symbioses across the auchenorrhynchan phylogeny. The popularization of DNA sequencing-based techniques enabled such investigation, but our knowledge is still restricted to a few Auchenorrhyncha clades, mainly within Cicadomorpha (12, 19, 20). While diagnostic screens revealed *Sulcia*, *Vidania*, and often other bacteria or fungi in most planthopper families (21), in only one species so far have nutritional symbionts been characterized using genomics. In the Hawaiian cixiid Oliarus filicicola, *Vidania* produces seven essential amino acids, and *Sulcia* synthesizes three, whereas a more recently acquired gammaproteobacterial symbiont, *Purcelliella*, contributes B vitamins (22). This rearrangement of *Sulcia* and *Vidania* nutritional responsibilities shed new light on the evolution of planthopper symbioses and how infections with additional microbes influence them. However, our understanding of symbioses in this diverse, widespread, and ecologically significant insect clade remains very limited.

This work aimed to provide an insight into the diversity and biology of symbioses in the large, diverse planthopper family Dictyopharidae. Previously, eight members of this family were shown to possess the bacteria *Vidania* and usually also *Sulcia* (21), but microscopic observations conducted by Müller (23, 24) and summarized by Buchner (14) indicated that they also host a third symbiont. They reported that these additional symbionts transmitted either together or separately from *Sulcia* and *Vidania*, suggesting their independent origins or other unusual phenomena, making this family a valuable system for the exploration of symbiont complementation and genome evolution.

Here, we report the results of microscopy and sequencing-based investigations of symbiotic bacteria associated with seven species belonging to the Dictyopharidae. We survey the diversity of the symbionts and explore their nutritional roles. In particular, we focus on how symbionts in this group are transmitted across generations. We describe and discuss how the auchenorrhynchan symbiont transmission can be separated in time and space, how this may have evolved, and what it may mean for the host.

## RESULTS

### Dictyopharidae planthoppers harbor (at least) three types of heritable symbionts.

**FIG 1:**
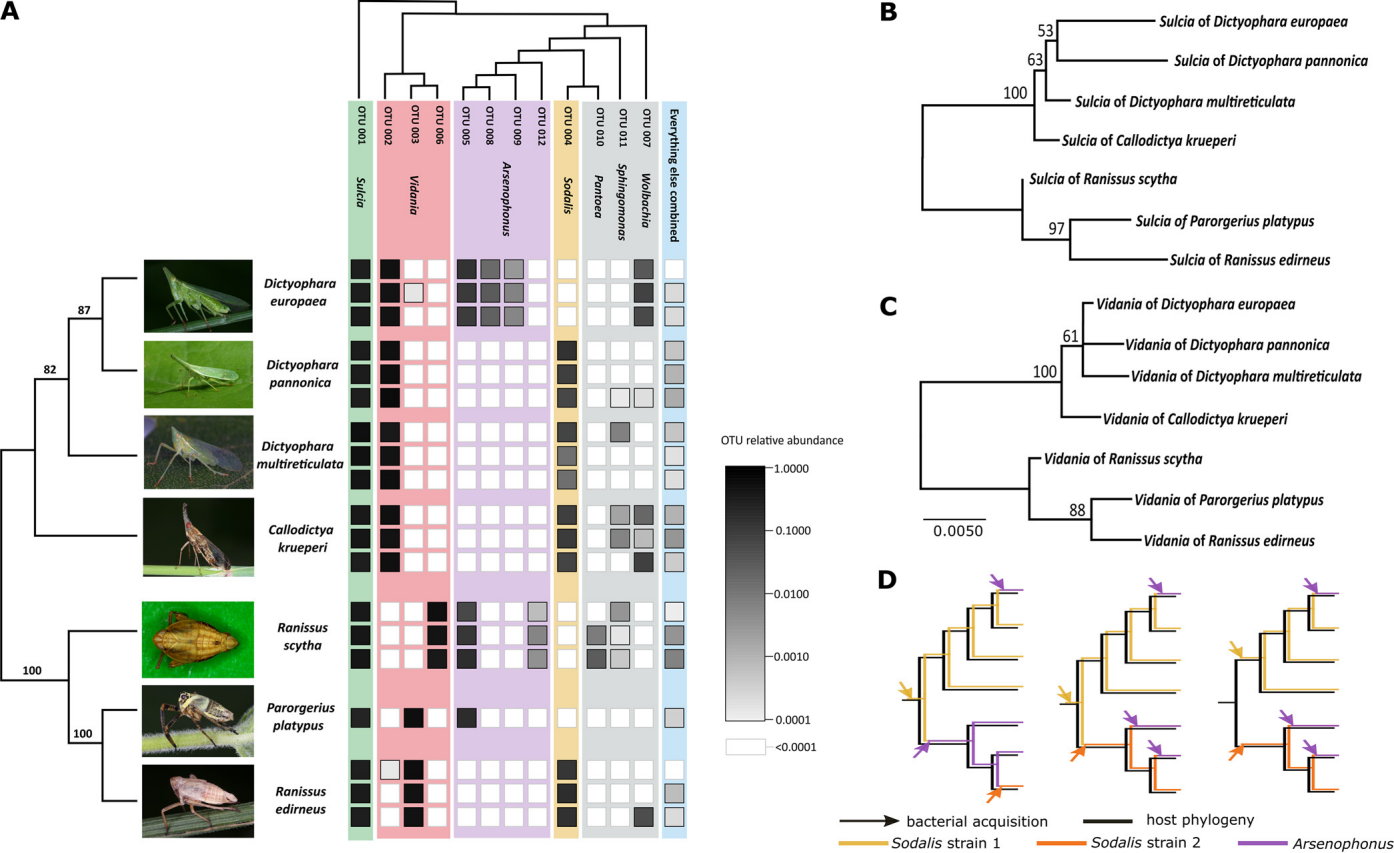
The bacterial communities in Dictyopharidae planthoppers. (A) The diversity and relative abundance of bacteria in replicate specimens from seven experimental species. Insect phylogeny (Maximum Likelihood [ML]) is based on concatenated marker gene sequences (COI, CytB, 18S rRNA, and 28S rRNA); bootstrap values are shown above the nodes. The bacterial tree is based on representative sequences of the 16S rRNA V4 region for different OTUs. (B and C) ML phylogenies for *Sulcia* and *Vidania* symbionts from seven experimental species, based on full-length 16S rRNA sequences. (D) Three of many possible scenarios of *Sodalis*/*Arsenophonus* acquisition and replacement during Dictyopharidae diversification.

Amplicon sequencing-derived mitochondrial cytochrome oxidase I (COI) sequences for the representative specimens of seven experimental species confirmed their morphology-based identifications (Fig. 1A; see also Table S3A and B in the supplemental material). The phylogeny revealed two well-supported clades corresponding to subfamilies Dictyopharinae and Orgerinae; it also showed that Ranissus edirneus is more closely related to Parorgerius platypus than to Ranissus scytha, in agreement with the proposed taxonomic revision of the subfamily Orgerinae (25). We successfully amplified the bacterial 16S rRNA gene V4 region from the abdomens of all experimental individuals from these seven Dictyopharidae species. The total number of 16S rRNA gene reads passed through all analysis steps was 452,949, or 23,840 per sample on average (Table S3C). Clustering with 97% identity cutoff identified 67 operational taxonomic units (OTUs).

10.1128/mBio.01228-21.7 TABLE S3 OTU tables. Download Table S3, XLSX file, 1.2 MB. Copyright © 2021 Michalik et al. 2021 Michalik et al. https://creativecommons.org/licenses/by/4.0/ This content is distributed under the terms of the Creative Commons Attribution 4.0 International license.

All studied individuals hosted *Sulcia* and *Vidania* and, additionally, either *Sodalis* or *Arsenophonus* (Fig. 1A and Table S3C). Together, these symbionts comprised >97% of reads in each of the libraries. The single-nucleotide-resolution data (Table S3D) for these dominant symbionts revealed high similarity among strains of the slow-evolving symbiont *Sulcia* genotypes and a greater distance among strains of *Vidania* from different Dictyopharidae clades, which grouped into three distinct 97% OTUs. Callodictya krueperi, Dictyophara multireticulata, Dictyophara pannonica, and *R. edirneus* hosted different genotypes of *Sodalis* (clustering to one OTU). D. europaea, *S. scytha*, and *P. platypus* hosted *Arsenophonus*, typically different genotypes from more than one OTU. However, the consistent relative abundance of genotypes in replicate individuals suggested that these genotypes/OTUs correspond to different rRNA operons within the genome of a single *Arsenophonus* strain (Fig. 1A). Less-abundant microbial OTUs present in some species included *Wolbachia*, *Pantoea*, and *Sphingomonas* (Fig. 1A). All the remaining OTUs combined accounted for 0.2% of the total number of reads, and many of them represented contaminants or symbiont-derived sequences that accumulated large numbers of errors. We did not consider them further.

**FIG 2:**
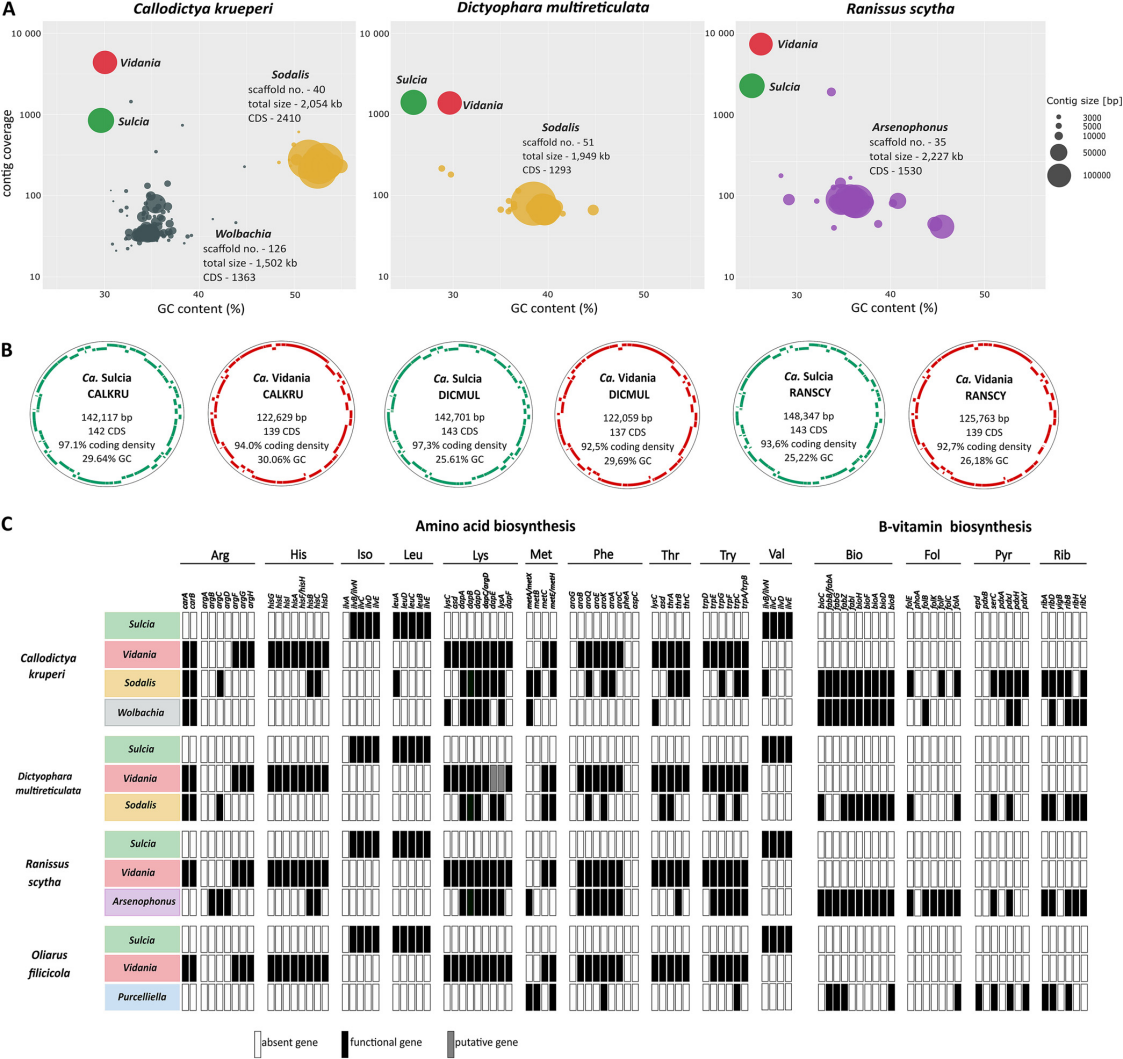
The summary of metagenomic data for three Dictyopharidae species. (A) Taxon-annotated GC content-coverage plots for metagenomic assemblies. Colored blobs represent scaffolds corresponding to the identified symbiont genome fragments. (B) Circular diagrams of *Sulcia* and *Vidania* genomes from three species, showing gene positions on forward and reverse strands and basic genome characteristics. (C) Contribution of symbionts to amino acid and B vitamin biosynthesis pathways. Standard abbreviations for amino acid, B vitamin, and gene names are used. Bio, biotin; Fol, folate; Pyr, pyridoxine; Rib, riboflavin.

**FIG 3:**
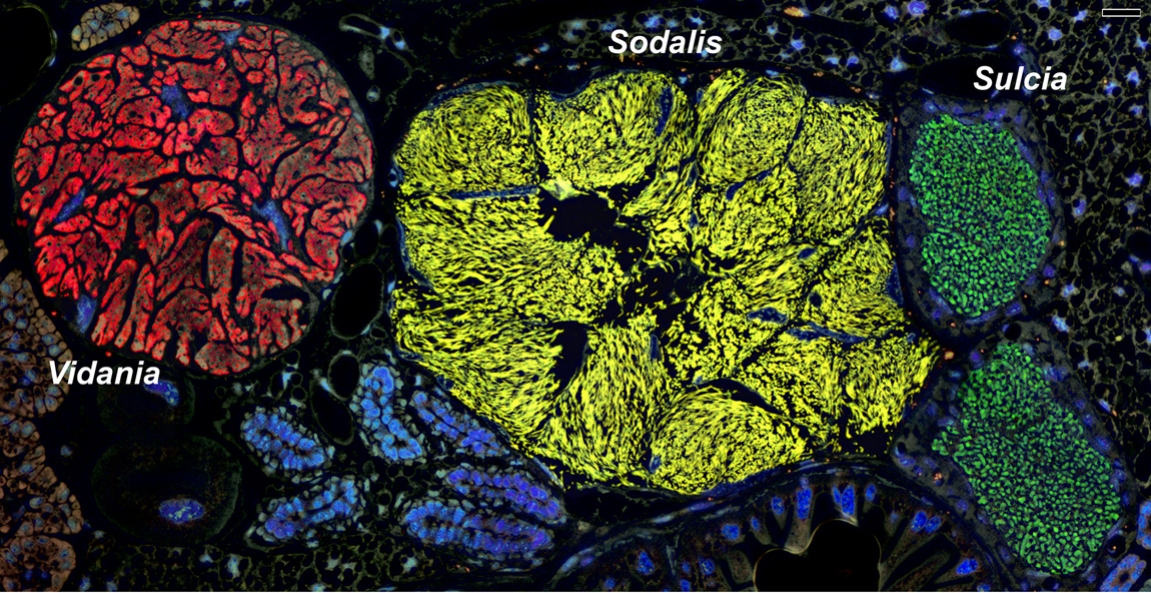
Fluorescence *in situ* hybridization (FISH) demonstrates how in *C. krueperi*, each of its three co-primary symbionts inhabits a distinct bacteriome. Transverse section through the insect’s abdomens. The image is oriented in dorsal-ventral position. Specific probes for *Vidania* (red), *Sulcia* (green), and *Sodalis* (yellow) were used. Blue represents cell nuclei stained with DAPI (4′,6-diamidino-2-phenylindole). Confocal laser microscope (CLM), bar = 20 μm.

**FIG 4:**
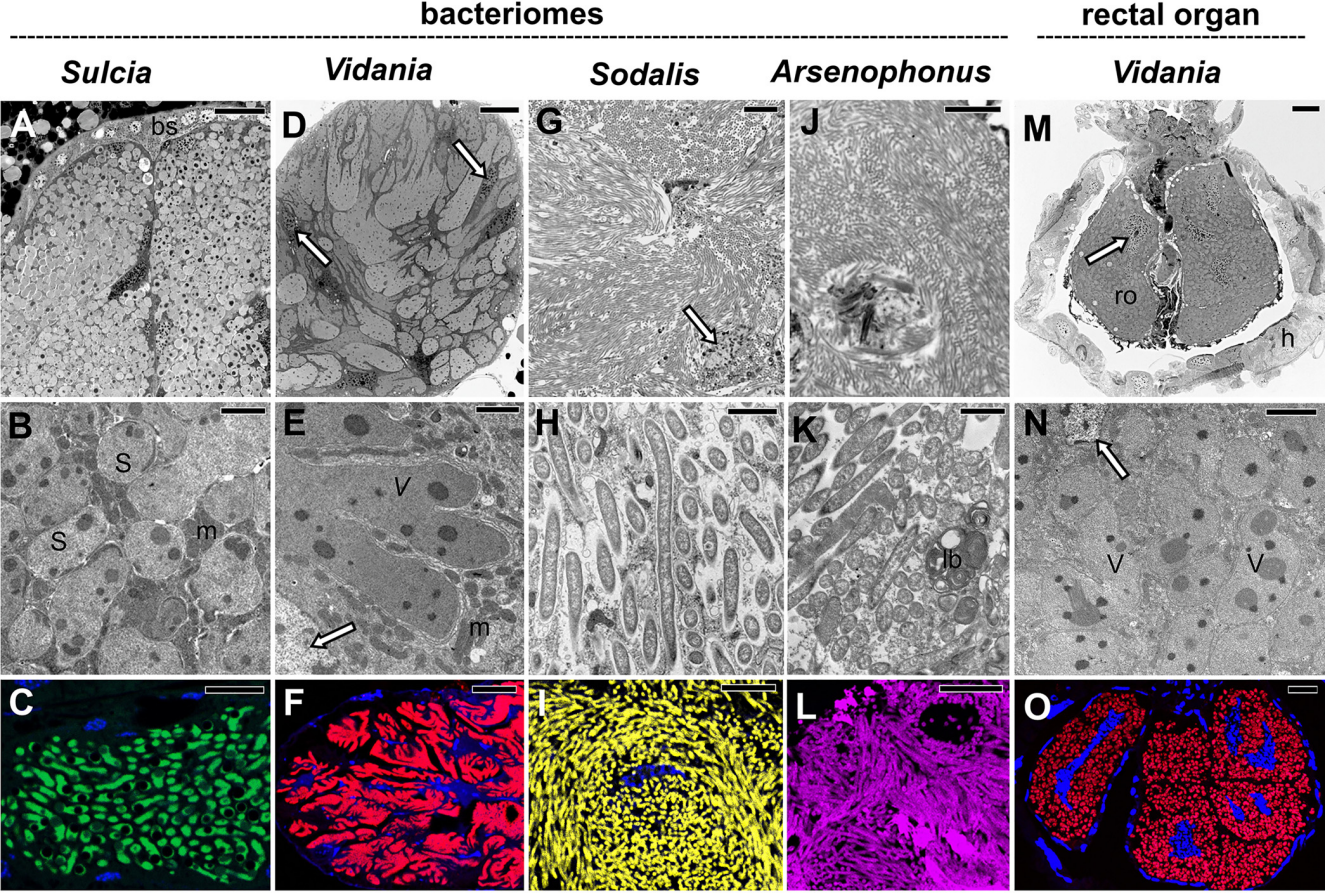
Tissue localization and morphology of symbionts in the Dictyopharidae species examined. Top row (A, D, G, J, and M): the organization of symbionts within bacteriomes or the rectal organ. Light microscopy (LM), bar = 20 μm. Middle row (B, E, H, K, and N): the ultrastructure of symbiont cells. Transmission electron microscopy (TEM), bar = 2 μm. Bottom row (C, F, I, L, and O): fluorescence *in situ* hybridization (FISH) microphotographs of symbiont cells within the bacteriome or rectal organ. Probes specific to each of the symbionts were used. Blue represents cell nuclei stained with DAPI. Confocal laser microscope (CLM), bar = 20 μm. Insect species: (A) *D. multireticulata*, (B and L) *R. scytha*, (C, E to I, M, and N) *C. krueperi*, (D and O) *D. pannonica*, (J and K) *D. europaea*. Abbreviations and symbol: bs, bacteriome sheath; h, hindgut; lb, lamellar body; m, mitochondrion; ro, rectal organ; S, *Sulcia*; V, *Vidania*; white arrow, bacteriocyte nucleus.

**FIG 5:**
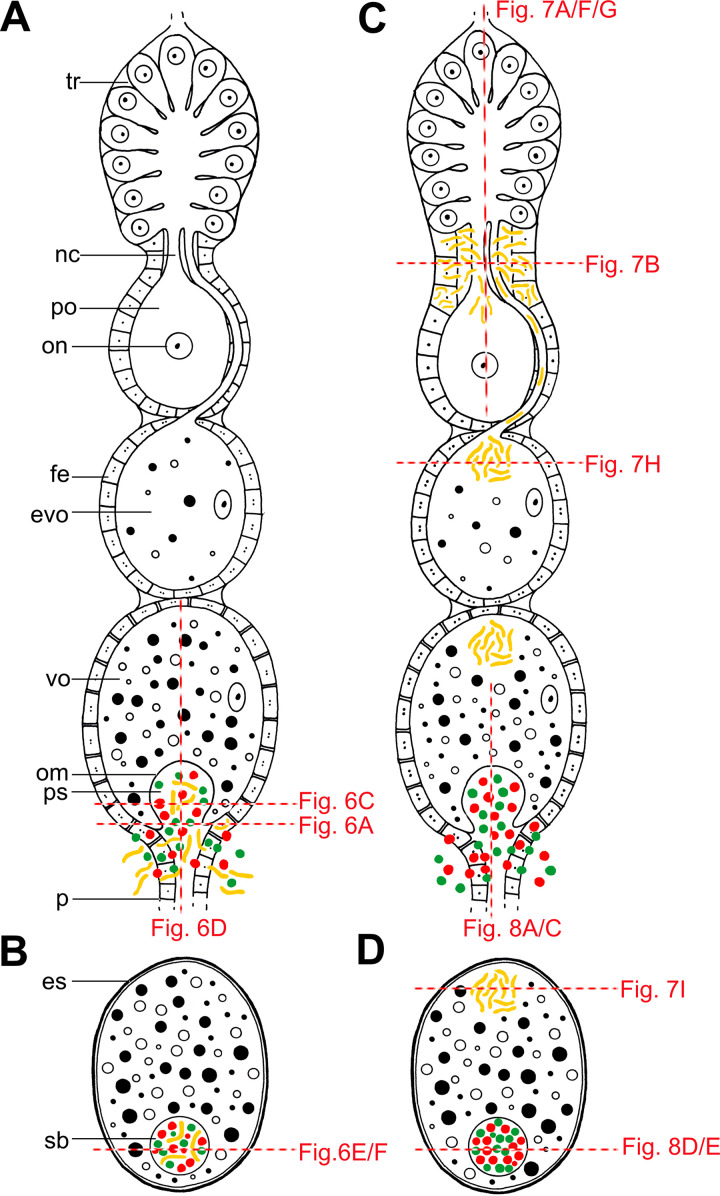
Schematic representation of the alternative modes of the symbiont transmission in Dictyopharidae planthoppers. (A) Simultaneous transmission of all types of nutritional symbionts through the follicular epithelium surrounding the posterior pole of terminal oocyte. (B) Full-grown oocyte with a “symbiont ball” containing three types of symbionts. The transmission mode shown in panels A and B is used by the large majority of Auchenorrhyncha, including subfamily Orgerinae and *Arsenophonus*-infected *D. europaea* from subfamily Dictyopharinae. (C) Spatially and temporally separated transmission of different symbionts (*Sodalis* versus *Sulcia* and *Vidania*). *Sodalis* infects previtellogenic and early-vitellogenic oocytes, whereas the remaining symbionts invade terminal vitellogenic oocytes. (D) Full-grown oocyte with the accumulation of *Sodalis* bacteria at the anterior pole and “symbiont ball” with *Sulcia* and *Vidania* cells at the posterior pole. This mode of transmission is unique to *Sodalis*-infected members of the subfamily Dictyopharinae. Red dashed lines indicate regions shown in panels of Fig. 6 to 8. Abbreviations: tr, tropharium; nc, nutritive cord; po, previtellogenic oocyte; on, oocyte nucleus; fe, follicular epithelium; evo, early-vitellogenic oocyte; vo, vitellogenic oocyte; om, oocyte membrane; p, pedicel; ps, perivitelline space; es, eggshells; sb, “symbiont ball.”

Phylogenetic trees of *Sulcia* and *Vidania* based on full-length 16S rRNA gene sequences (Fig. 1B and C) are congruent with the phylogeny of the seven experimental Dictyopharidae species (Fig. 1A), or a broader range of planthoppers (Fig. S1A and B), as expected for symbionts codiversifying with hosts. In contrast, 16S rRNA gene phylogenies for *Sodalis* and *Arsenophonus* from diverse hosts, while poorly resolved and supported, were suggestive of independent origins of at least some of the cosymbionts of Dictyopharidae (Fig. S1C and D). This hypothesis was further supported using phylogenomics: phylogenies reconstructed based on 129 conserved single-copy protein-coding genes of symbiotic enterobacteria and relatives, constructed using reference alignments and following the methods of McCutcheon and colleagues (18) and including *C. krueperi*, *D. multireticulata*, and *R. scytha* symbionts, strongly suggest that these three symbionts originated from independent infections (Fig. S1E). These data, combined with information on the distribution of the two symbiont clades across the host phylogeny and differences in transmission patterns and genomics characteristics (described below), indicate several independent infections by *Arsenophonus*/*Sodalis* or their repeated replacements by other strains of these symbionts (Fig. 1B to D and Fig. 2 to 5). Unfortunately, the currently available data do not allow for a reconstruction of the order of these infections. Three of many possible scenarios, all assuming relatively few replacement events and shared ancestry of some strains, are presented in Fig. 1D. However, the number of independent infections or replacements along the tree branches could have been much greater. In fact, patterns such as the 16S rRNA amplicon genotype diversity across *D. pannonica* individuals (Table S3D) and some morphological differences (Fig. S3A) among *Sodalis* cell populations hint at the possibility of recent or perhaps ongoing replacements.

10.1128/mBio.01228-21.2 FIG S1 ML phylogenies of symbionts based on 16S rRNA gene sequences. (A) ML phylogeny of *Sulcia* strains from diverse planthoppers, based on full-length sequences (1,373 shared nucleotide positions) of 16S rRNA gene. Only bootstrap values above 70% are shown. Sequences from the seven Dictyopharidae species studied here are highlighted using the colored font. (B) ML phylogeny of *Vidania* strains from diverse planthoppers based on full-length sequences (1,530 shared nucleotide positions) of 16S rRNA gene. Only bootstrap values above 70% are shown. Sequences from the seven Dictyopharidae species studied here are highlighted using the colored font. (C) ML phylogeny of *Sodalis* strains from different hosts based on 16S rRNA gene sequences. Only bootstrap values above 70% are shown. (D) ML phylogeny of *Arsenophonus* strains from different hosts based on 16S rRNA gene sequences. Only bootstrap values above 70% are shown. (E) ML phylogeny based on first and second codon positions of 129 conserved single-copy protein-coding genes of symbiotic enterobacteria and relatives. Only bootstrap values above 70% are shown. Download FIG S1, PDF file, 1.8 MB. Copyright © 2021 Michalik et al. 2021 Michalik et al. https://creativecommons.org/licenses/by/4.0/ This content is distributed under the terms of the Creative Commons Attribution 4.0 International license.

10.1128/mBio.01228-21.4 FIG S3 (A) *Dictyophara pannonica*. Two morphotypes of *Sodalis* symbiont (red and yellow arrows) within bacteriome of single individuals. TEM, bar = 2 μm. (B) *Wolbachia* symbiont cells within bacteriocytes of Dictyopharidae species (TEM). (1) *Wolbachia* cells (green arrows) in the cytoplasm of the *Vidania* (V) bacteriocyte from *Callodictya krueperi*. (2) *Wolbachia* cells (green arrows) in the cytoplasm of the *Sulcia* (S) bacteriocyte from *Dictyophara europaea*. TEM, bar = 2 μm. Download FIG S3, PDF file, 1.1 MB. Copyright © 2021 Michalik et al. 2021 Michalik et al. https://creativecommons.org/licenses/by/4.0/ This content is distributed under the terms of the Creative Commons Attribution 4.0 International license.

### *Sulcia* and *Vidania* provide essential amino acids to host insects, whereas *Arsenophonus*, *Sodalis*, and *Wolbachia* supplement their diet with B vitamins.

The symbiont genomes identified in the assemblies matched those identified by amplicon sequencing (Fig. 2A). The circular genomes of *Sulcia* and *Vidania* ranged in size between 142 and 148 kb and 122 to 125 kb, respectively, and were characterized by relatively low GC contents (25 to 30%) and high coverage (800 to 10,000×) (Fig. 2B). *Sulcia* and *Vidania* genomes were colinear relative to each other and the slightly larger reference genomes of *O. filicicola* (OLIH) symbionts (157 and 136 kb, respectively) (Fig. S2) (22). Our metagenomic analyses also confirmed the presence of *Sodalis* and *Wolbachia* in *C. krueperi*, *Sodalis* in *D. multireticulata*, and *Arsenophonus* in *R. scytha*. The genomic assemblies of these symbionts were fragmented; the number of scaffolds ranged between 23 and 126 and their total size between 1,500 kb and 2,220 kb. The scaffold read coverage was substantially lower than in the cases of *Sulcia* and *Vidania* but also relatively variable, particularly in the case of *Arsenophonus* (Table S4A).

10.1128/mBio.01228-21.3 FIG S2 The alignments of planthopper *Sulcia* and *Vidania* genomes. (A) PROMER alignments of final *Sulcia* genomes from three Dictyopharidae species, *Callodictya krueperi* (CALKRU), *Dictyophara multireticulata* (DICMUL), and *Ranissus scytha* (RANSCY), against the *Sulcia* genome from *Oliarus filicicola* (OLIH; family Cixiidae). (B) PROMER alignments of final *Vidania* genomes from three Dictyopharidae species, *Callodictya krueperi* (CALKRU), *Dictyophara multireticulata* (DICMUL), and *Ranissus scytha* (RANSCY), against the *Vidania* genome from *Oliarus filicicola* (OLIH; Cixiidae). Download FIG S2, PDF file, 0.1 MB. Copyright © 2021 Michalik et al. 2021 Michalik et al. https://creativecommons.org/licenses/by/4.0/ This content is distributed under the terms of the Creative Commons Attribution 4.0 International license.

10.1128/mBio.01228-21.8 TABLE S4 Genome details. Download Table S4, XLSX file, 0.3 MB. Copyright © 2021 Michalik et al. 2021 Michalik et al. https://creativecommons.org/licenses/by/4.0/ This content is distributed under the terms of the Creative Commons Attribution 4.0 International license.

In terms of gene content, the newly characterized *Sulcia* and *Vidania* genomes are very similar to each other and the previously described OLIH symbiont genomes (22). *Sulcia* genomes include 142 to 143 predicted protein-coding genes, 27 to 29 identifiable tRNAs, and complete ribosomal operon, and their coding density ranges from 93.6% to 97.3%. *Vidania* genomes include 137 to 139 predicted protein-coding genes, 23 to 25 tRNAs, and complete ribosomal operon, with coding density ranging between 92.5% and 94% (Fig. 2B). In the two analyzed *Sodalis* genomes, Prokka identified 2,410 (CALKRU) and 1,132 (DICMUL) predicted protein-coding genes; in *Arsenophonus*, 1,530; and in *Wolbachia*, 1,363. However, because of the incompleteness of the assemblies of these cosymbiont genomes and challenges with pseudogene annotation, these numbers are approximate.

*Sulcia* and *Vidania* complement each other in provisioning host insects with 10 essential amino acids, in a way consistent among the three Dictyopharidae and OLIH (Fig. 2C). *Sulcia* participates in the biosynthesis of three essential amino acids including isoleucine, leucine, and valine, whereas *Vidania* is involved in synthesizing the remaining seven. Interestingly, some of the biosynthetic pathways (isoleucine, arginine, methionine, phenylalanine) were not complete, with genes missing from *Sulcia* and *Vidania* genomes (Fig. 2C). Some of these missing amino acid biosynthesis genes, and duplicate copies of some others, are present in *Sodalis*, *Arsenophonus*, and *Wolbachia* genomes. For example, in *C. krueperi*, *Vidania* lacks the first two genes essential for methionine biosynthesis from homoserine, but both are present in the *Sodalis* genome. Similarly, in *D. multireticulata*, *Sodalis* complements two pseudogenes (*dapE*, *lysA*) in the *Vidania* genome, part of the lysine biosynthesis pathway. *Arsenophonus* from *R. scytha* has the same set of phenylalanine and tryptophan biosynthesis genes as *Vidania*. In all Dictyopharidae, the gammaproteobacterial symbionts contain many genes in the lysine biosynthesis pathway and scattered genes from other pathways, and the list might get extended once assemblies are complete.

In addition to genes involved in amino acids’ biosynthesis, *Sodalis*, *Arsenophonus*, and *Wolbachia* contribute to the synthesis of B vitamins: biotin, folate, riboflavin, and pyridoxine (Fig. 2C). *Sodalis* and *Wolbachia* associated with *C. krueperi* as well as *Arsenophonus* of *R. scytha* contain full sets of biotin biosynthesis genes, whereas the genome of *Sodalis* of *D. multireticulata* carries 8 of 10 genes from that pathway. The *Arsenophonus* symbiont genome also carries the majority of genes involved in the biosynthesis of folate and riboflavin. Interestingly, in *C. krueperi*, both *Sodalis* and *Wolbachia* appear capable of riboflavin biosynthesis.

### Nutritional symbionts of dictyopharids occupy distinct but adjacent bacteriocytes.

In all dictyopharids studied, nutritional symbionts (*Sulcia*, *Vidania*, and either *Sodalis* or *Arsenophonus*) reside within separate bacteriomes located close to each other within the insect abdomen (Fig. 3 and 4). Light microscopy observations revealed that bacteriomes harboring bacteria *Sulcia*, *Sodalis*, and *Arsenophonus* are made up of several bacteriocytes (Fig. 3 and 4A), whereas bacteriomes with *Vidania* are syncytial (Fig. 3 and 4D). Bacteriomes occupied by *Sulcia* and *Vidania* are surrounded by a thick or thin monolayered bacteriome sheath, respectively (Fig. 4A, C, D, and F). In contrast, bacteriomes with *Arsenophonus* and *Sodalis* are not covered by epithelial cells (Fig. 3).

Our histological, ultrastructural, and fluorescence *in situ* hybridization (FISH) analyses revealed that *Sulcia* cells are pleomorphic and possess large electron-dense inclusions in their cytoplasm (Fig. 3 and 4A to C). *Vidania* cells are giant and multilobed, with numerous electron-dense accumulations in the cytoplasm (Fig. 3 and 4D to F). The cells of gammaproteobacterial symbionts *Sodalis* and *Arsenophonus* are large, elongated, and morphologically similar in all host species (Fig. 3 and 4J to L). In particular, we did not observe distinct *Arsenophonus* morphotypes, further supporting our amplicon-based conclusions that different 16S OTUs correspond to different rRNA operons in the genome of a single strain (Fig. 4J to K).

In all species analyzed, bacteriocytes have large, polyploid nuclei, and their cytoplasm is tightly packed with symbionts, ribosomes, and mitochondria (Fig. 3 and 4). The density of mitochondria in bacteriocytes with *Sulcia* and *Vidania* seemed substantially higher than in bacteriocytes with *Sodalis* and *Arsenophonus*. *Sulcia* and *Vidania* tightly adhere to each other in the cytoplasm of their bacteriocytes, whereas *Sodalis* and *Arsenophonus* are somewhat less densely packed (Fig. 3 and 4G to L). Finally, ultrastructural observations in *C. krueperi* and *D. europaea* revealed small, rod-shaped bacteria in the cytoplasm of bacteriocytes housing *Sulcia* and *Vidania* (Fig. S3B). Their shape and size matched *Wolbachia*, detected in these species using amplicon sequencing.

Besides bacteriomes, *Vidania* also occupies bacteriocytes in the rectal organ, localized in the invagination of hindgut epithelium (Fig. 4M to O). *Vidania* cells in the rectal organ differ in shape and size from *Vidania* localized in the bacteriomes.

### Dictyopharids have developed different symbiont transmission strategies.

**FIG 6:**
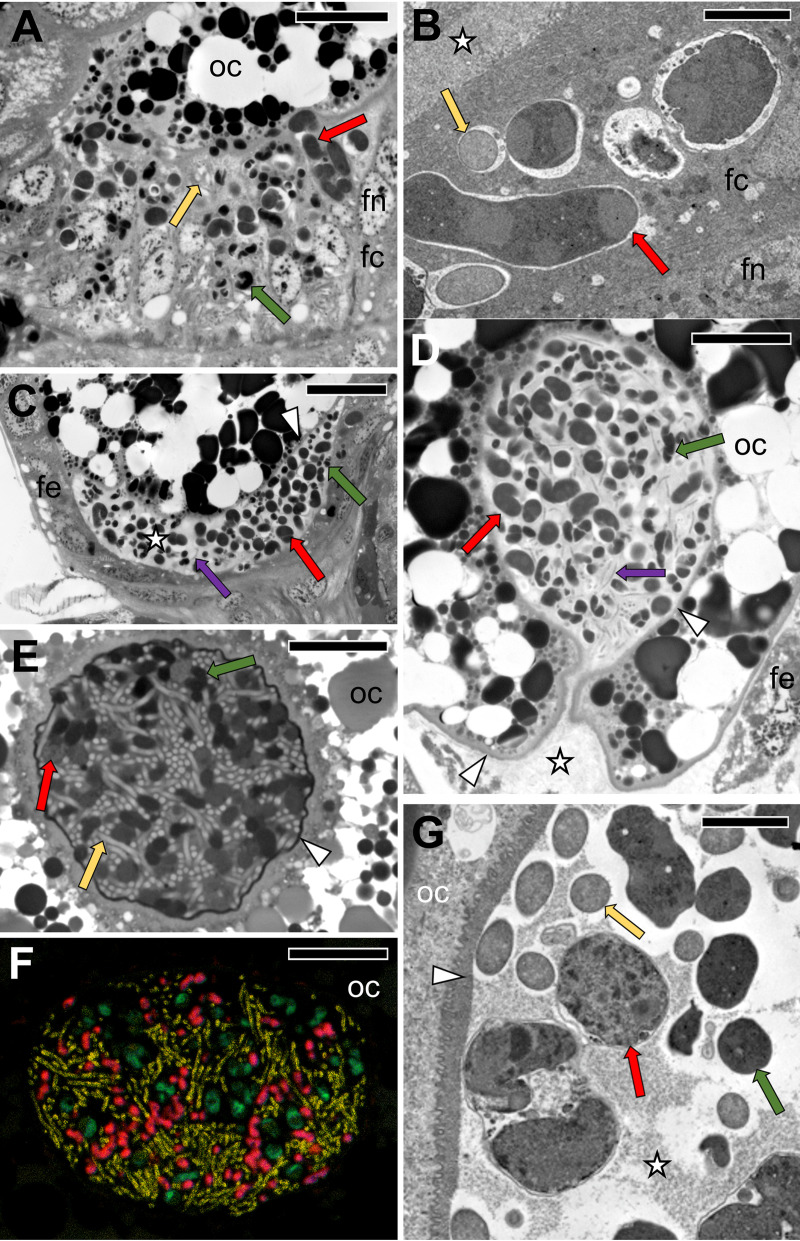
The simultaneous transmission of three symbionts in selected Dictyopharidae, as shown in Fig. 5A and B. (A) The migration of *Sulcia*, *Vidania*, and *Arsenophonus* through follicular cells surrounding the posterior pole of the terminal oocyte. *D. europaea*, light microscopy (LM), bar = 20 μm. (B) *Sodalis* and *Vidania* in the cytoplasm of the follicular cell. *R. edirneus*, TEM, bar = 2 μm. (C and D) Symbiotic bacteria in the perivitelline space. *D. europaea*, LM, bar = 20 μm. (E) A “symbiont ball” containing bacteria *Sulcia*, *Vidania*, and *Sodalis* in the deep depression of the oolemma at the posterior pole of the terminal oocyte. *R. edirneus*, LM, bar = 20 μm. (F) *In situ* identification of symbionts in the “symbiont ball” within the terminal oocyte. *R. edirneus*, CLM, bar = 20 μm. (G) Fragment of the “symbiont ball” in the perivitelline space. *R*. *edirneus*, TEM, bar = 2 μm. Abbreviations and symbols: fc, follicular cell; fn, the nucleus of the follicular cell; oc, oocyte; asterisk, perivitelline space; arrowhead, oocyte membrane; red cells/arrows, *Vidania*; green cells/arrows, *Sulcia*; yellow cells/arrows, *Sodalis*; purple arrows, *Arsenophonus*.

**FIG 7:**
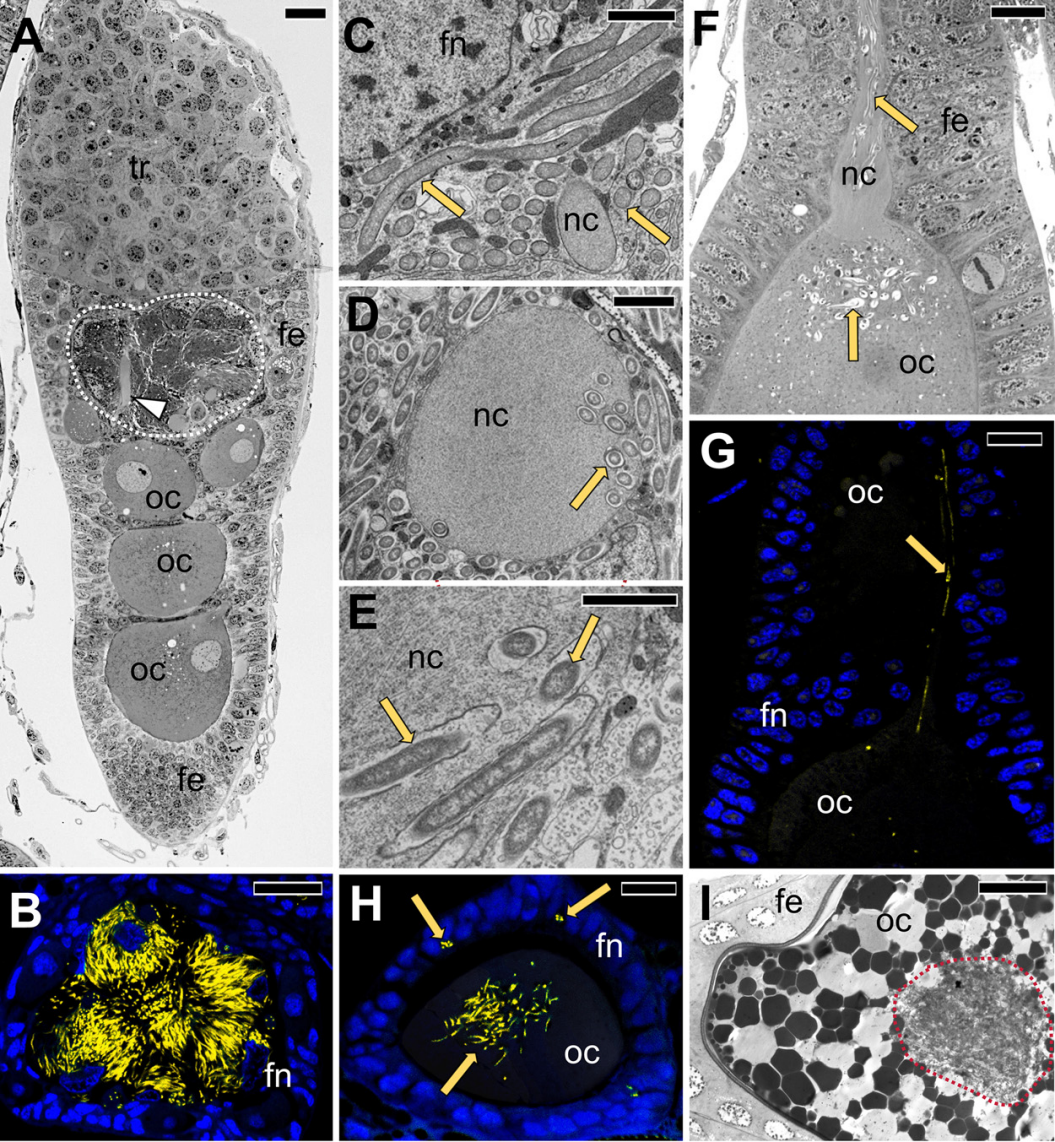
Transovarial transmission of *Sodalis* into the anterior pole of the developing oocyte in representatives of Dictyopharinae subfamily. (A) Longitudinal section through the ovariole in the previtellogenesis stage. Note numerous *Sodalis* cells in the region of the ovariole between tropharium and vitellarium (area surrounded with white dotted line). *D. pannonica*, LM, bar = 20 μm. (B) Cross-section through the neck region of the ovariole filled with prefollicular cells with bacteria *Sodalis* (marked in yellow); *C. krueperi*, confocal microscope, bar = 20 μm. (C) Fragment of the prefollicular cell with *Sodalis* occupying the neck region of the ovariole. *D. multireticulata*, TEM, bar = 2 μm. (D) Cross-section through the nutritive cord surrounded by *Sodalis*. Note *Sodalis* cells in the cytoplasm of the nutritive cord. *C. krueperi*, TEM, bar = 2 μm. (E) The higher magnification of *Sodalis* bacteria migrating via nutritive cord to the oocyte. *C. krueperi.* TEM, bar = 2 μm. (F and G) The transport of *Sodalis* via nutritive cord to the previtellogenic oocyte. *C. krueperi.* (F) LM, bar = 20 μm. (G) CLM, bar = 20 μm. (H) Accumulation of *Sodalis* cells in the cytoplasm of the anterior region of the previtellogenic oocyte. *C. krueperi*, CLM, bar = 20 μm. (I) Accumulation of *Sodalis* in the cytoplasm of the choriogenic oocyte. *D. multireticulata*, LM, bar = 20 μm. Abbreviations and symbol: fe, follicular epithelium; fn, the nucleus of a follicular cell; nc or arrowhead, nutritive cord; oc, oocyte; yellow cells/arrows, *Sodalis*.

**FIG 8:**
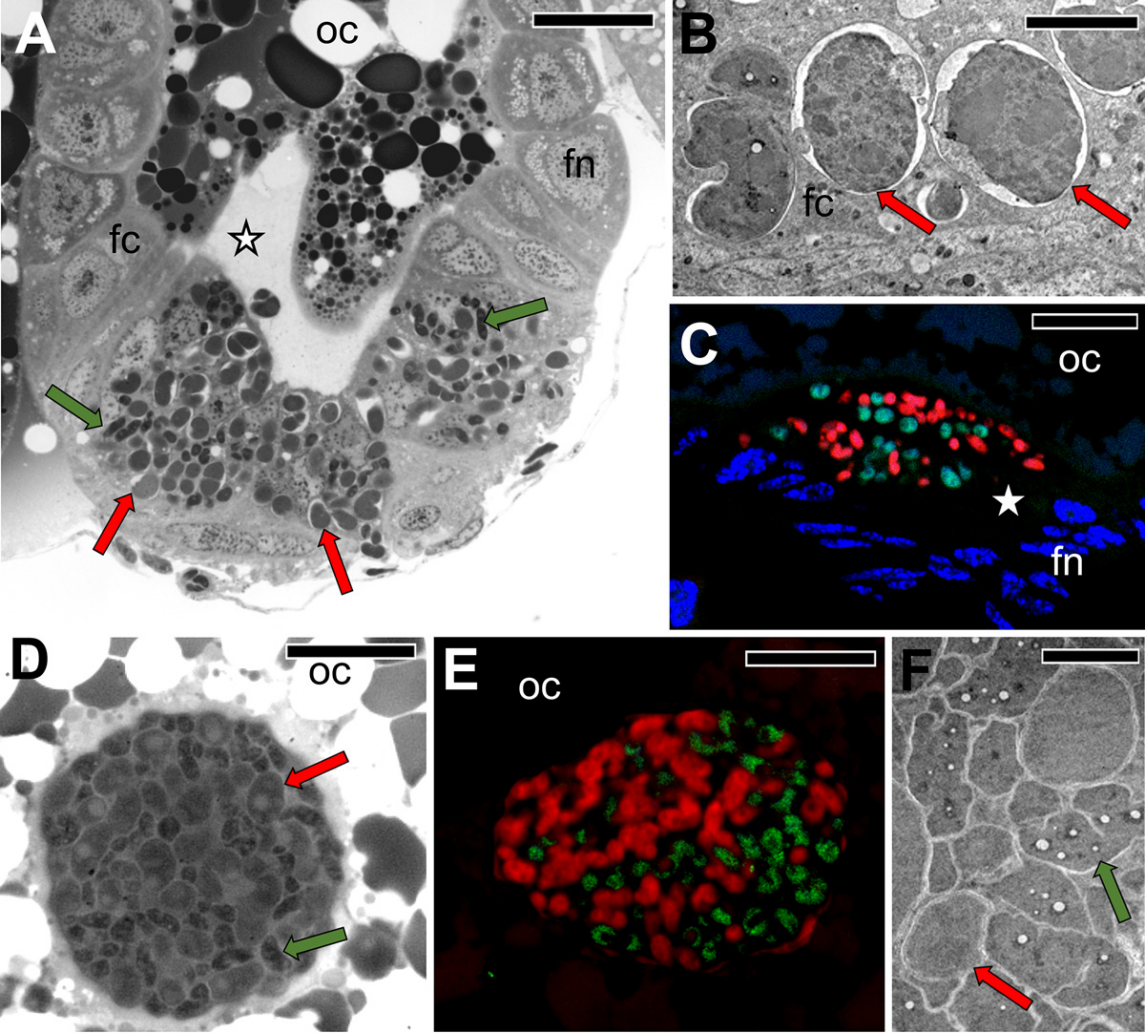
Transovarial transmission of *Sulcia* and *Vidania* into the posterior end of the ovariole in representatives of Dictyopharinae subfamily. (A) The migration of *Sulcia* and *Vidania* to the perivitelline space through the follicular epithelium surrounding the posterior pole of the terminal oocyte. *D. multireticulata*, LM, bar = 20 μm. (B) *Vidania* in the cytoplasm of the follicular cell. *D. pannonica*, TEM, bar = 2 μm. (C) Accumulation of *Sulcia* (green) and *Vidania* (red) in the perivitelline space. *C. krueperi.* Confocal microscope, bar = 20 μm. (D) A “symbiont ball” containing *Sulcia* and *Vidania* in the deep depression of the oolemma at the posterior pole of the terminal oocyte. *D. multireticulata*, LM, bar = 20 μm. (E) *In situ* identification of symbionts in the “symbiont ball” in the mature oocyte of *C. krueperi*. CLM, bar = 20 μm. (F) Fragment of “symbiont ball.” *D. multireticulata*, TEM, bar = 2 μm. Abbreviations and symbols: fc, follicular cell; fn, the nucleus of a follicular cell; oc, oocyte; asterisk, perivitelline space; red cells/arrows, *Vidania*; green cells/arrows, *Sulcia*.

Histological observations of serial semithin sections revealed that nutritional symbionts of the studied Dictyopharidae are transmitted between generations transovarially. The details of *Sulcia*, *Vidania*, and *Arsenophonus* transmission agree with observations from other Auchenorrhyncha. Strikingly, we observed significant differences in the transmission of *Sodalis* (Fig. 5 to 8).

In all species examined, *Sulcia* and *Vidania* simultaneously migrate and infect the posterior end of the ovariole during the choriogenesis stage of oocyte development (Fig. 5A and Fig. 6A). Symbionts migrate to the perivitelline space (=space between follicular epithelium and oolemma) via the cytoplasm of follicular cells surrounding the posterior pole of the terminal oocyte (Fig. 5, Fig. 6A and B, and Fig. 8A and B). These follicular cells are enlarged during symbiont migration, and their cytoplasm is full of bacterial cells (Fig. 6A and Fig. 8A). *Arsenophonus* (in *D. europaea*, *R. scytha*, and *P. platypus*) and *Sodalis* (in *R. edirneus*) are transmitted together with ancestral symbionts (Fig. 6A to D). After passing the follicular epithelium, symbionts gather in the perivitelline space. First, they create a cup-like accumulation (Fig. 6C) and then form a symbiont ball in the deep invagination of the oolemma (Fig. 6D to G).

In contrast, in *C. krueperi*, *D. pannonica*, and *D. multireticulata*, we observed the separation of symbiont transmission in time and space (Fig. 5C). Instead of entering the perivitelline space through follicular cells, *Sodalis* infects the region of the ovariole between the tropharium and vitellarium. We observed large accumulations of *Sodalis* cells in ovarioles containing oocytes in the previtellogenic stage of oogenesis. The follicular cells separating the tropharium from the vitellarium are tightly packed with bacteria (Fig. 7A to C). This specific region of the ovariole is penetrated by nutritive cords which connect oocytes developing in the vitellarium with trophocytes localized in the tropharium (Fig. 7A to C). *Sodalis* then infects previtellogenic oocytes using nutritive cords (Fig. 7D to H). They leave the follicular cells *en masse*, enter the nutritive cord area (Fig. 7D and E), and migrate toward the oocyte along microtubules (Fig. 7F to H). Then, *Sodalis* cells aggregate in the cytoplasm of the anterior pole of the oocyte (Fig. 7H) and stay in this form through the next stages of oogenesis (Fig. 7I). In these species, *Sulcia* and *Vidania* symbionts are transmitted to the perivitelline space of choriogenic oocytes (which contain clusters of *Sodalis* bacteria in the cytoplasm of the anterior pole) separately and, as summarized before, through follicular cells at their posterior end (Fig. 8A to C). They closely adhere to each other and form a characteristic “symbiont ball” (Fig. 8D to F).

In contrast to *Sulcia*, *Sodalis*, and *Arsenophonus*, which do not change their shape substantially during migration, *Vidania* undergoes significant morphological changes. In comparison to lobate *Vidania* cells within bacteriocytes, migrating *Vidania* cells (in the cytoplasm of follicular cells and in the “symbiont ball”) are smaller and more spherical (Fig. 6 and 8), resembling those occupying the rectal organ.

## DISCUSSION

### Repeated symbiont replacements shape the symbiosis in Dictyopharidae planthoppers.

Amplicon sequencing, metagenomics, and microscopy data agreed that all studied species of Dictyopharidae host heritable nutritional symbionts *Sulcia* and *Vidania*. We know that *Sulcia* infected the common ancestor of all Auchenorrhyncha that lived about 300 MYA, and *Vidania*, together with spittlebug-associated *Zinderia* and leafhopper-associated *Nasuia*, appears to represent a similarly ancient lineage (2, 17, 26). We found surprisingly few differences in the organization or contents of genomes of these widely retained symbionts (14, 21, 23, 24) between Dictyopharidae and the only other planthopper studied using genomics, *O. filicicola* OLIH, even though they are separated by about 200 MY of evolution (2). Dictyopharidae symbiont genomes are somewhat smaller than those in OLIH (142 to 148 kb versus 156 kb for *Sulcia*, 122 to 126 kb versus 136 kb for *Vidania*), but they are colinear and carry essentially the same set of nutrient biosynthesis genes. *Sulcia* CALKRU has the most compact genome of many strains of this symbiont characterized so far. *Vidania* falls among bacteria with the smallest known genomes, alongside other hemipteran symbionts: *Nasuia* (≥110 kb), *Tremblaya* (≥138 kb), *Carsonella* (≥164 kb), and *Hodgkinia* (≥144 kb, but note that individual genomes in fragmented complexes can be much smaller) (7, 27–30).

The reduction of symbiont genomes in long-lasting symbiotic interactions is a natural consequence of the lack of DNA repair genes or recombination, allowing for the progressive accumulation of deleterious mutations (31). Combined with the increase in rates of evolution, this leads to rapid loss of genes and pathways engaged in metabolic processes redundant during intracellular life and is thought to negatively affect the function and efficiency of others, including those involved in essential cellular processes as well as biosynthesis of nutrients (32–34). This opens up a path to their complementation or replacement by newly arriving, more versatile microorganisms. From this evolutionary perspective, both the complementation and replacement of “old” symbionts by “new” ones should allow host insects to refresh and reset their biosynthetic capacity and likely positively influence fitness and perhaps extend their ecological niche (17, 18). The radiation of insect clades whose ancestors acquired new symbionts, including cicadas, sharpshooter leafhoppers, aphids, carpenter ants, and others, is suggestive of a competitive edge that the nutritional symbiont acquisition or replacement can provide (35, 36).

In addition to the two ancestral symbionts, all studied Dictyopharidae harbor heritable, bacteriocyte-associated enterobacteria, either *Sodalis* or *Arsenophonus*, apparently acquired several times independently. These two microbial clades have repeatedly infected diverse insects, assuming nutritional functions especially in sap- and blood-feeders (2, 37–40). *Arsenophonus* can also act as a facultative symbiont, reproductive manipulator, or insect-vectored plant pathogen (2, 41–43). In Auchenorrhyncha, *Sodalis* and *Arsenophonus* are usually associated with ancestral symbionts, presumably complementing them (40). For example, they have been reported alongside ancestral symbionts in leafhoppers Macrosteles laevis and Cicadella viridis and in the spittlebug Aphrophora quadrinotata (10, 12, 44). Other times, they appear to have replaced one of the ancient symbionts. For example, the loss of *Zinderia* and the acquisition of *Sodalis* in modern Philaenini spittlebugs seems to be linked (12). There is little information on how often such replacements happen, but similarly to what was suggested in mealybugs (28), serial replacements are a likely explanation for the diversity of *Sodalis*/*Arsenophonus* symbionts within the studied Dictyopharidae. We will get a much more complete picture of the incidence and significance of this symbiont complementation and replacement as we survey gamma-symbiont distributions more systematically and use larger numbers of whole genomes for phylogenetic reconstruction events (17, 18).

### The remarkable conservation of tissue localization and functions in dynamic planthopper symbioses.

In all Dictyopharidae species analyzed, as in other planthoppers examined previously, the ancestral symbionts *Sulcia* and *Vidania* are localized in distinct, spatially separated bacteriomes within the insect body cavity (14, 45–47). This contrasts with the situation in Cicadomorpha, where the ancestral symbionts always inhabit the same bacteriome: *Sulcia*, as a rule, occupies bacteriocytes within the outer layer of the bacteriome, whereas the coresiding ancestral symbiont (*Nasuia*, *Zinderia*, or *Hodgkinia*) occupies the central portion (11, 14, 29, 48). The differences in the spatial organization of symbiont-housing organs may be due to chance but could also be related to their mutual relations and nutritional capabilities. In Cicadomorpha, co-primary symbionts are thought to share some cofactors and metabolites, mainly involved in the biosynthesis of energetically expensive amino acids such as methionine or histidine (4), and this could be facilitated by the proximity of bacteriocytes inhabited by different symbionts.

Gammaproteobacterial symbionts, including *Arsenophonus* and *Sodalis*, localize differently in different insect hosts. The gammaproteobacterial symbionts of Dictyopharidae, despite their varied origins, are always located within bacteriomes separated from, but adjacent to, those occupied by *Sulcia* and *Vidania*. In other Auchenorrhyncha, they can also occur in separate bacteriomes or, alternatively, colonize distinct bacteriocytes within existing bacteriomes, but they are also observed in the cytoplasm of the same bacteriocytes as ancestral symbionts, or even inside other symbionts’ cells (10, 11, 20). In other insects, they may be dispersed across host tissues other than bacteriocytes, including gut epithelium cells, fat body cells, and hemolymph (49, 50). Interestingly, in some systems, independently acquired microbes tend to inhabit the same tissue compartments, suggestive of preadaptations that make it a particularly hospitable place. A striking example is gammaproteobacterial symbionts of Pseudococcinae mealybugs, always residing inside the cells of the ancient *Tremblaya* symbiont (28).

In females of all Dictyopharidae, similarly to previously studied planthopper species, we observed two morphotypes of *Vidania* occupying distinct bacteriomes in the body cavity and rectal organ. Buchner (14) and Bressan and Mulligan (46) suggested that symbionts derived from the rectal organ are the infectious form of *Vidania* that are transmitted to the progeny. Future transcriptomic studies should clarify the roles of these morphotypes, but our microscopic observations agree with the view that *Vidania* cells which are transmitted to the ovary represent the rectal organ morphotype.

In Cicadomorpha, *Sulcia* carries genes involved in the biosynthesis of seven to eight amino acids, while the companion symbiont is responsible for the provisioning of the remaining two or three. In Fulgoromorpha, the relative contributions of the companion symbionts are reversed. The ability to synthesize arginine, lysine, phenylalanine, and threonine has been lost by *Sulcia*, and they are produced by *Vidania* instead. Notably, the nearly identical biosynthetic capabilities in Dictyopharidae and the divergent cixiid, *O. filicicola*, reveal how stable their ancestral nutritional symbionts can be over 200 MY of evolution alongside changing gammaproteobacterial partners. The consistent differences in *Sulcia* biosynthetic capabilities between Cicadomorpha and Fulgoromorpha suggest that the two clades have separated soon after the acquisition of the ancestral beta-symbiont, assuming that there was indeed one, as proposed but not unambiguously demonstrated (17). Later, stochastic or other factors must have caused differential gene and pathway loss among the partner symbionts in the ancestors of the two infraorders.

In planthoppers, at least those studied to date, B vitamin biosynthesis has been outsourced to additional gammaproteobacterial symbionts, for example, *Purcelliella* in *O. filicicola* or *Arsenophonus* in Nilaparvata lugens (22, 51). Indeed, all characterized planthopper-associated *Sodalis* and *Arsenophonus* strains possess largely complete sets of genes involved in riboflavin and biotin production. Additionally, *Sodalis* of *C. krueperi* and *Arsenophonus* of *R. scytha* may be able to synthesize pyridoxine and folate, respectively. A similar biosynthetic potential was found in *Sodalis*-related symbionts of mealybugs (28) and *Arsenophonus* living in symbiotic relations with other insects such as the wasp Nasonia vitripennis and many whiteflies and louse flies (39, 40, 52). Moreover, both *Sodalis* and *Arsenophonus* possess some genes from essential amino acid biosynthesis pathways. Most of them overlap those retained on the *Vidania* genome, but some complement *Vidania* in methionine and lysine production. Such biosynthetic pathway complementarity between the host and symbionts has been demonstrated experimentally in the mealybug system (18) but is likely to be a more universal phenomenon (28, 29). It would be interesting to explore more broadly whether there is a general trend toward complementarity, how it is affected by serial co-primary symbiont replacements, and whether it can facilitate the replacement of ancient symbionts.

### Newly arriving symbionts utilize different strategies for transovarial transmission.

The crucial role of nutritional symbionts in insect biology is evidenced by the diversity of their vertical transmission routes (15, 16). Transovarial transmission is probably the most reliable means of providing the full symbiont complement to all offspring. In Auchenorrhyncha, ancient symbionts are transmitted in a consistent, conserved way, crossing the follicular cells surrounding the posterior pole of the vitellogenic oocytes and then forming the “symbiont ball” near its posterior end (16). Additional symbionts generally follow the same path (11, 48, 53), including *Arsenophonus* and *Sodalis* in some of the Dictyopharidae species presented here. In others, *Sodalis* has adopted a very different transmission strategy, invading the ”neck region” of the ovariole from where it is carried to the cytoplasm of previtellogenic oocytes via the nutritive cords. Müller (23, 24) and Buchner (14) emphasized that the diversity of strategies that newly arriving insect symbionts adopt help us understand the general patterns of symbiosis evolution and replacement. The fact that closely related microbes have adopted different strategies in related host species is particularly notable. Nonetheless, the spatiotemporal separation of transovarial symbiont transmission is not unique to this insect clade. For example, in the planthopper Cixius nervosus, one of its symbionts, unidentified so far, was observed to infect undifferentiated cystocytes, while others infect full-grown oocytes (16).

In other groups of hemipterans and more divergent insects, gammaproteobacterial nutritional symbionts have adopted an even wider range of transovarial transmission strategies. For instance, in some scale insects, bacteria invade larval ovaries and germ cells at a very early stage, before they differentiate into oocytes and trophocytes, and are later present in all germ cells in the ovariole (16). In some heteropteran bugs but also carpenter ants, symbiotic gammaproteobacteria infect early previtellogenic oocytes directly: infect follicular cells, gather in their cytoplasms, and then enter the oocyte’s cytoplasm via an endocytic pathway. In both cases, initially, symbionts are dispersed in the entire ooplasm, but during vitellogenesis they accumulate and form a “symbiont ball” (54, 55). In some host lineages, enterobacterial symbionts have adopted more exotic transmission strategies. In *Cicadella viridis* and *Macrosteles laevis*, gammaproteobacterial symbionts (*Sodalis* and *Arsenophonus*, respectively) live inside and are moved to the oocyte within *Sulcia* cells (10, 44). In the scale insect Puto superbus, whole, intact bacteriocytes containing *Sodalis* are transferred into ovarioles (56). In turn, in tsetse flies, Sodalis glossinidius is vertically carried into the developing larva via milk gland secretions (57).

Fitness differences between these alternative transmission strategies are not apparent. It is likely that they vary in efficiency, including in the proportion of cells departing from the bacteriomes that arrive within the oocytes, or the energy needed for each symbiont cell to complete the journey. It is also probable that there are differences in the maintenance costs of the mechanisms necessary to move the cells between bacteriomes and oocytes and, later, to the target regions of the developing embryo. Transporting gammaproteobacterial symbionts along the same path as *Sulcia* and *Vidania* could theoretically allow the host to reuse the existing cellular machinery in a relatively efficient manner but that would depend on the specificity of the mechanisms, something that we have virtually no knowledge of. Yet, another aspect relevant to fitness, especially in the long run, is the symbiont bottleneck size, which may affect the strength of genetic drift and the symbiont evolutionary rates (53). However, at the moment, we know little about the magnitude of any such differences or how important they might be. But ultimately, and most critically, all mechanisms appear to result in reliable transmission of all symbionts to every offspring.

Why do some symbionts adopt different ways of transmission than others, then? We suspect that this is a combination of preadaptations, existing constraints, and chance events. The transmission strategy, but also final tissue localization, could be related to the microbe’s biology prior to the transition to the endosymbiotic lifestyle. The presence of specific genes or pathways in the microbe’s genome, and mechanisms that direct them toward, or facilitate entry into, certain cell types, is likely to drive the differences (58). At the same time, the reutilization of existing host-encoded mechanisms, including those for the transmission of older symbionts, is likely to play a significant role, probably increasing over time as symbiont genomes degenerate and erode away. For example, *Spiroplasma* and *Wolbachia* were shown to utilize the receptors involved in the transport of vitellogenin (protein precursors of yolk) into oocytes to enable entry into developing eggs (59, 60). We suspect that stochastic processes—random changes affecting the affinity between host and symbiont structural molecules at early stages of infection—play a significant role in determining the way the symbionts end up transmitting.

### Conclusions.

The analyses of multipartner associations in Hemiptera provide unique insight into the factors shaping the evolution of nutritional symbiosis, highlighting the evolutionary significance and broad relevance of symbiont replacements which happened repeatedly in planthoppers and other hemipterans (12, 17, 19, 28, 61–64). While the list of nutritional symbionts that have reached a relatively stable stage and remained largely unchanged for millions or hundreds of millions of years extends beyond the familiar names such as *Sulcia*, *Buchnera*, *Blochmannia*, *Baumannia*, etc., it is also becoming clear that this is not a universal fate for newly acquired symbionts. Even related host species may harbor independently acquired symbiont strains with genomes at various stages of genomic degradation. It suggests that the “symbiont acquisition—genomic degradation—replacement” cycle is more frequent than often thought (17, 19, 63). This means that newly arriving microbes regularly face the challenge of establishing residence within host tissues and developing effective means of transmission across host generations. While there are differences among host clades in the organization of the symbiont-containing tissue, replacing symbionts often arrive at the same location within the host. Moreover, they generally undergo similar genome-reductive processes and converge on the same roles. Yet despite many common features, the detailed strategies utilized by the replacing symbionts may vary. Alternative strategies of intergenerational symbiont transmission in Dictyopharidae planthoppers are a key example. Our understanding of factors determining how the choice of strategies adopted by new symbionts, preadaptations, and random chance influence the outcomes of new infections is extremely limited. However, in the world of heritable nutritional symbioses that is proving unexpectedly dynamic, with many symbionts going through a rapid cycle of serial symbiont replacements, the balance between deterministic and stochastic factors is likely to determine the outcome of many symbioses.

## MATERIALS AND METHODS

### Study species.

We investigated symbioses in seven planthopper species from two subfamilies within the family Dictyopharidae. From subfamily Dictyopharinae, we characterized four species: *Callodictya krueperi* (Fieber, 1872), *Dictyophara pannonica* (Germar, 1830), *Dictyophara multireticulata* (Mulsant & Rey, 1855), and *Dictyophara europaea* (Linnaeus, 1767). From Orgerinae, we studied three species: *Ranissus* (*Schizorgerius*) *scytha* (Oshanin, 1913), *Ranissus* (*Ranissus*) *edirneus* (Dlabola, 1957), and *Parorgerius platypus* (Fieber, 1866). Adult specimens were sampled from a single population of each species in either Bulgaria or Poland between 2016 and 2018 (see Table S1 in the supplemental material), preserved in 80% ethanol or glutaraldehyde, and stored at 4°C until processing. Representative specimens from each species were identified based on morphological characteristics, and identities were confirmed using marker gene sequencing.

10.1128/mBio.01228-21.5 TABLE S1 Collection details. Download Table S1, XLSX file, 0.01 MB. Copyright © 2021 Michalik et al. 2021 Michalik et al. https://creativecommons.org/licenses/by/4.0/ This content is distributed under the terms of the Creative Commons Attribution 4.0 International license.

### Amplicon-based microbiome screen.

**(i) Library preparation and sequencing.** We sequenced amplicons for the V4 hypervariable region of the bacterial 16S rRNA gene and host mitochondrial cytochrome oxidase I (COI) genes simultaneously. DNA extracted using the Bio-Trace extraction kit (Eurx, Gdańsk, Poland) from dissected insect abdomens for up to three individual insects per species (plus negative controls) was used for amplicon library preparations following a modified two-step preparation protocol (65). In the first round of PCR, we amplified two marker regions of interest, using template-specific primers 515F/806R (66, 67) and COIBF3/COIBR2 (68) with Illumina adapter stubs. The bead-purified PCR products were used as the template for the second, indexing PCR. Pooled libraries were sequenced on an Illumina MiSeq v3 lane (2 × 300-bp reads) at the Institute of Environmental Sciences of Jagiellonian University. The primer sequences and detailed protocols are provided in Table S2.

10.1128/mBio.01228-21.6 TABLE S2 Protocols. Download Table S2, XLSX file, 0.02 MB. Copyright © 2021 Michalik et al. 2021 Michalik et al. https://creativecommons.org/licenses/by/4.0/ This content is distributed under the terms of the Creative Commons Attribution 4.0 International license.

**(ii) Analyses of amplicon sequencing data.** We processed 16S rRNA and COI gene amplicon data using mothur v. 1.43.0 (69) following a pipeline detailed in Text S1. Initially, all amplicon data sets were split into bins corresponding to the two target genes. For both bins, we assembled reads into contigs. Quality-filtered, dereplicated contigs, with singletons removed, were aligned against the reference databases, screening 16S rRNA gene alignments for chimeras using UCHIME and then classifying sequences taxonomically. Finally, the sequences were clustered at 97% identity level using the nearest-neighbor algorithm and divided into OTUs.

10.1128/mBio.01228-21.1 TEXT S1 Bioinformatic pipelines for the analysis of COI and 16S rRNA amplicon data. Download Text S1, DOCX file, 0.02 MB. Copyright © 2021 Michalik et al. 2021 Michalik et al. https://creativecommons.org/licenses/by/4.0/ This content is distributed under the terms of the Creative Commons Attribution 4.0 International license.

### Metagenomic library preparation and sequencing.

We sequenced bacteriome metagenomic libraries for three species: *C. krueperi* (specimen ID: CALKRU), *D. multireticulata* (DICMUL), and *R. scytha* (RANSCY). DNA from dissected bacteriomes of individual insects, extracted using the Sherlock AX kit (A&A Biotechnology, Gdynia, Poland), was fragmented using a Covaris E220 sonicator and used for metagenomic library preparation using the NEBNext Ultra II DNA Library Prep kit for Illumina (New England BioLabs), with the target insert length of 350 bp. The library pool, including three target species and other samples, was sequenced on an Illumina HiSeq X SBS lane by NGXBio (San Francisco, CA, USA).

### Metagenome characterization and symbiont genome annotation.

Metagenomic reads were quality filtered using the ‘iu-filter-quality-minoche’ program included in illumina-utils software v1.4.4 (70) with default parameters (71). Contigs were assembled using Megahit v1.2.9 (k-mer 255, min contig size 1,000) (72). Because of the known issue of index swapping that occurs during cluster formation and sequencing on Illumina platforms (73, 74), which can lead to cross-contamination among samples in multiplexed lanes, we filtered the resulting assemblies for cross-contamination: we discarded all contigs that had >10× (and typically >200×)-greater coverage estimated based on strictly mapped reads from another library with one out of two indices shared than based on reads from the same library.

We identified symbiont contigs using BLASTN and tblastx searches against a custom database containing genomes of multiple known insect symbionts, verifying the identifications using coverage and GC content information (computed using BBTools v. 38.78). Then, for *Sulcia* and *Vidania* contigs, we confirmed their circularity and contiguity by read mapping and visualization on Tablet v. 1.20.12.24 (75) and the presence of overlapping ends. We rearranged the circularized genomes to ensure the same orientation and start position as in those published previously. *Arsenophonus*, *Sodalis*, and *Wolbachia* genomes were represented by multiple contigs, and we did not attempt to close them.

The genomic contigs of the three latter symbionts were annotated using Prokka v.1.14.6 (76), with the default parameters. For *Sulcia* and *Vidania*, Prokka but also InterProScan and GhostKOALA annotation attempts left multiple obvious gaps and unannotated or hypothetical proteins, and hence, we decided to use a custom workflow, modified from reference 29. Annotation was conducted by recursive searches for a manually curated set of alignments of protein-coding, rRNA, and noncoding RNA (ncRNA) genes from previously characterized genomes using HMMER 3.1b2 (77). Any open reading frames of at least 300 nucleotides that had not been annotated by the script were manually searched using hmmer and blastx/tblastx against UniProt and NCBI databases. All genes annotated as “hypothetical” or unannotated were carefully manually compared against the top hits in other microorganisms using blastp (https://blast.ncbi.nlm.nih.gov) and HMMER 3.3 (https://www.ebi.ac.uk/Tools/hmmer), resulting in the discovery of additional genes. Reference-based annotations of rRNA genes were supplemented by rRNA searches using RNAmmer v. 1.2 (78) and tRNA searches using tRNAscan-SE v. 1.4 (79). For all genes, we aligned all copies classified as functional using mafft v. 7.221 (80). In the case of protein-coding genes, alignments were conducted in protein space and reverse translated to nucleotide space.

The taxon-annotated GC-coverage plots for symbiont contigs were drawn using R v. 4.0.2 (R Development Core Team) with the ggplot2 package (81). Genomes were visualized using DNAPlotter GUI. In order to reconstruct amino acid and B vitamin biosynthetic pathways, we translated circular *Sulcia* and *Vidania* genomes as well as *Sodalis*, *Arsenophonus*, and *Wolbachia* contigs to amino acids and annotated them against KEGG with GhostKOALA (genus_prokaryotes) (82). After that, the presence of genes involved in biosynthetic pathways was checked manually.

### Cloning, amplification, and phylogenetic and phylogenomic analyses.

For the four species for which metagenomes were not sequenced (*D. pannonica*, *D. europaea*, *R. edirneus*, and *P. platypus*), we obtained full-length 16S rRNA gene sequences of symbionts through molecular cloning in Escherichia coli cells as described previously (47). We also PCR amplified planthopper mitochondrial *CytB* and nuclear 18S and 28S rRNA genes (Table S2). Purified PCR products were Sanger sequenced by Genomed S.A. (Warsaw, Poland). Trimmed reads were merged into contigs and aligned, and alignments were inspected using CodonCode Aligner v. 8.0.2 (CodonCode Corp., Centerville, MA, USA).

We conducted phylogenetic analyses of concatenated insect marker genes and bacterial 16S rRNA genes, including those obtained from metagenomes using MEGA 7 software (83), using the Maximum Likelihood algorithm assuming the GTR (for insect genes) and GTR+GAMMA (for bacterial genes) models and with 1,000 bootstrap replicates.

Phylogenomic analyses of gammaproteobacterial symbiont genomes closely followed methods described in the supplement of a recent review that provided a genome-based phylogeny for 93 enterobacterial symbionts and their relatives (63). We used curated alignments of 129 conserved single-copy protein-coding genes provided by the authors and HMMER to identify the top homolog in the annotation for the three Dictyopharidae symbionts and mafft to align all sequences in amino acid space. We used RAxML v.8 (84) to reconstruct the phylogeny based on first and second codon positions in the concatenated alignment set, specifying GTR+GAMMA as the model of evolution, and with 100 bootstraps computed as support.

### Microscopy.

**(i) Light (LM) and electron (TEM) microscopy.** Partially dissected abdomens of females of each of the seven species were fixed in the field in 2.5% glutaraldehyde solution in 0.1 M phosphate buffer (pH 7.4). In the laboratory, after washing with the same buffer with the addition of sucrose (5.8%), they were postfixed in a 1% solution of osmium tetroxide, dehydrated in ethanol and acetone series, and embedded in epoxy resin Epon 812 (Serva, Heidelberg, Germany). For histological analyses, the resin blocks were cut into serial, semithin sections, stained in 1% methylene blue in 1% borax, and observed under the Nikon Eclipse 80i light microscope. Ultrathin sections for ultrastructural analyses were contrasted with lead citrate and uranyl acetate and observed under the JEOL JEM 2100 electron transmission microscope.

**(ii) Fluorescence microscopy.** For fluorescence *in situ* hybridization (FISH), insects preserved in 80% ethanol were rehydrated and postfixed in 4% paraformaldehyde for 2 h. Then, the material was dehydrated again in the increasing concentration of ethanol and acetone and embedded in Technovit 8100 resin (Kulzer, Wehrheim, Germany). Resin blocks were cut into semithin sections and hybridized overnight at room temperature with symbiont-specific probes (Table S2). After hybridization, the slides were washed in phosphate-buffered saline (PBS), dried, covered with ProLong Gold antifade reagent (Life Technologies), and examined using a confocal laser scanning microscope, Zeiss Axio Observer LSM 710.

### Data availability.

Accession numbers for sequences of the planthopper symbionts discussed in this paper may be found in Table S5 in the supplemental material.

10.1128/mBio.01228-21.9 TABLE S5 Accession numbers. Download Table S5, XLSX file, 0.07 MB. Copyright © 2021 Michalik et al. 2021 Michalik et al. https://creativecommons.org/licenses/by/4.0/ This content is distributed under the terms of the Creative Commons Attribution 4.0 International license.

10.1128/mBio.01228-21.10 TABLE S6 Custom database. Download Table S6, XLSX file, 0.01 MB. Copyright © 2021 Michalik et al. 2021 Michalik et al. https://creativecommons.org/licenses/by/4.0/ This content is distributed under the terms of the Creative Commons Attribution 4.0 International license.
