# Supplementary material for: Alternative Transmission Patterns in Independently Acquired Nutritional Cosymbionts of Dictyopharidae Planthoppers
Source: mBio. 2021 Aug 31;12(4):e01228-21. doi: 10.1128/mBio.01228-21 (PMC8406288; doi:10.1128/mBio.01228-21)
Supplement: FIG S1 [file mbio.01228-21-sf001.pdf]

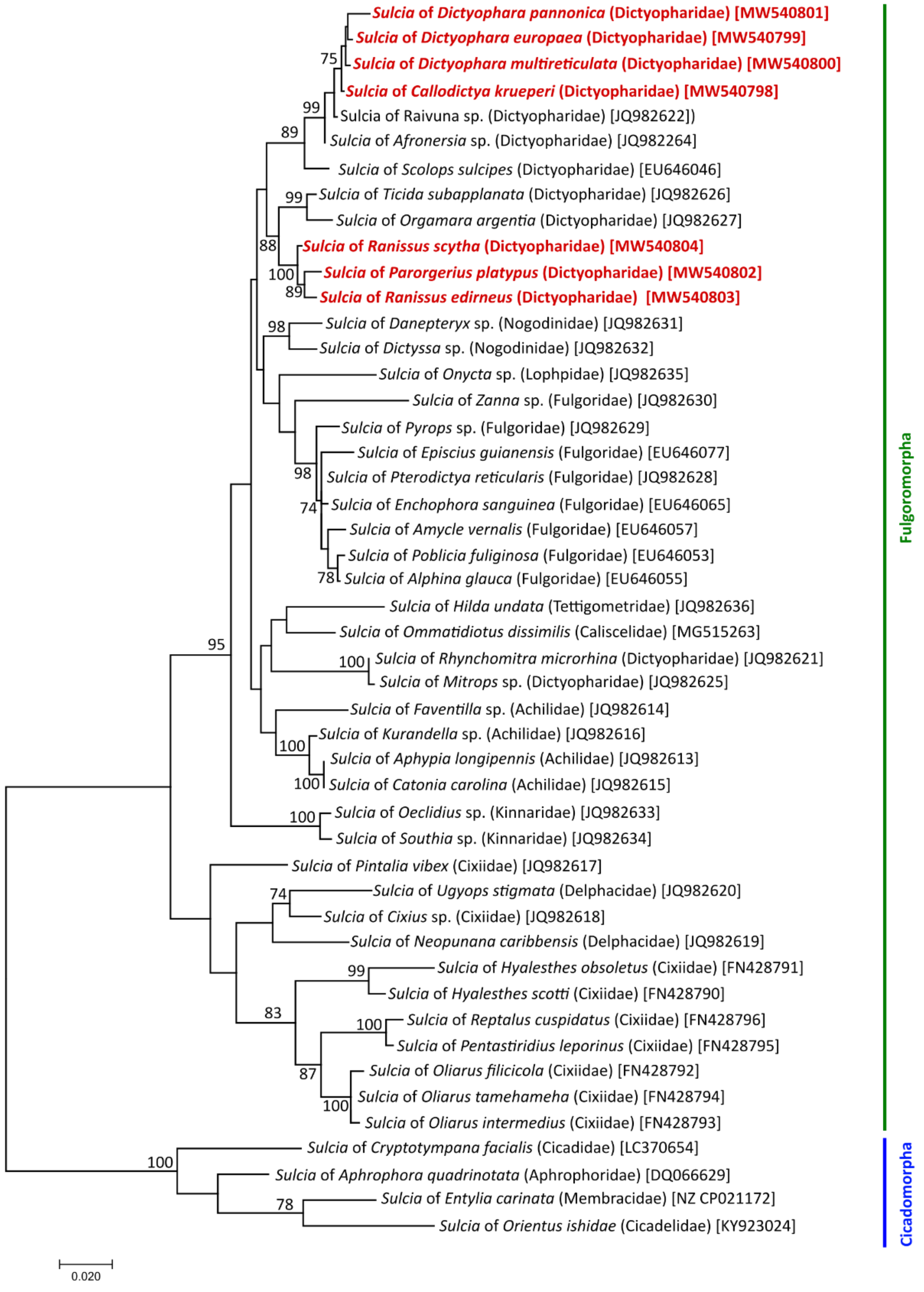

Fig. S1A. ML phylogeny of *Sulcia* strains from diverse planthoppers, based on full-length sequences (1373 shared nucleotide positions) of 16S rRNA gene. Only bootstrap values above 70% are shown. Sequences from the seven Dictyopharidae species studied here are highlighted using the colored font.

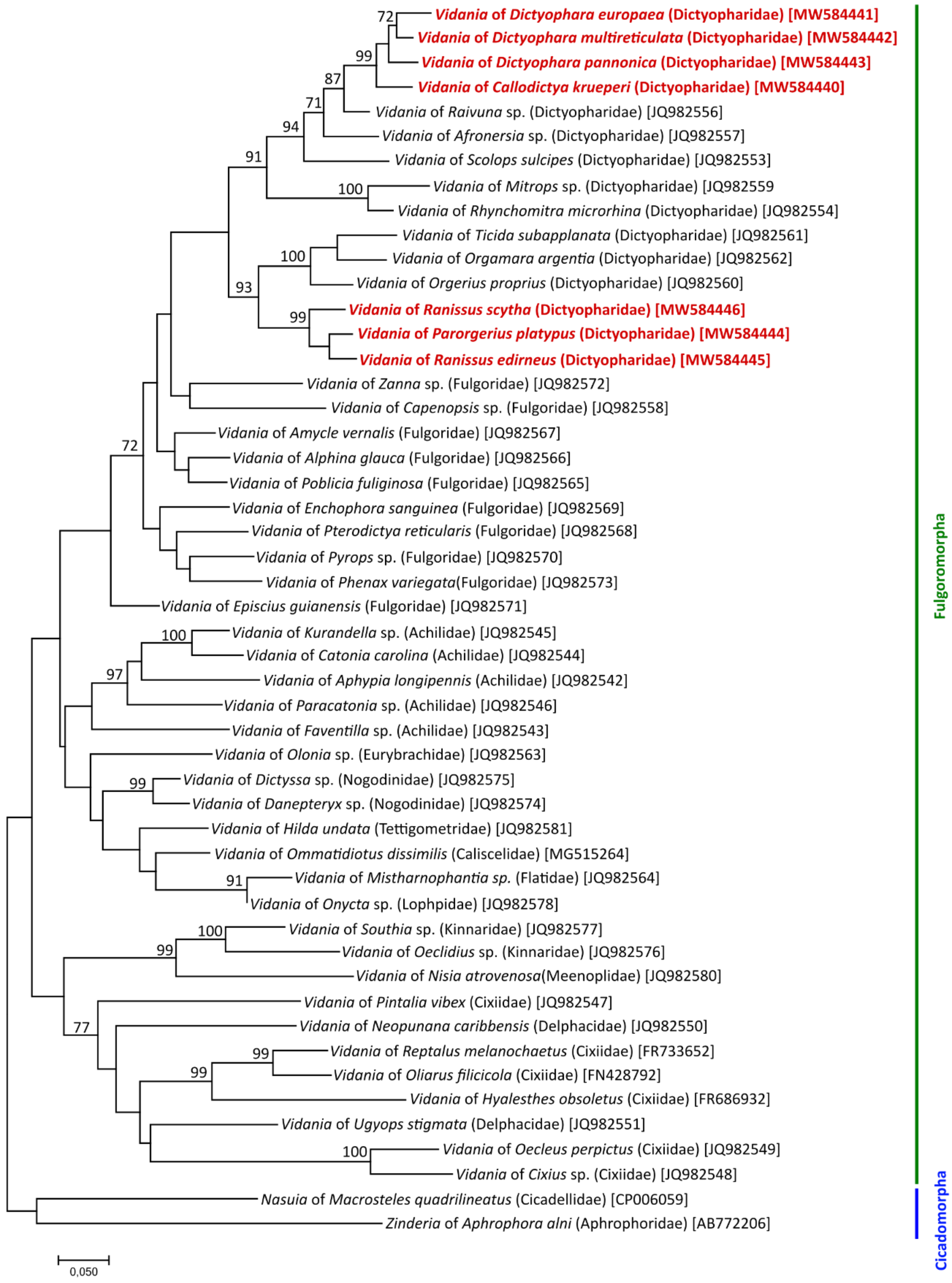

Fig. S1B. ML phylogeny of *Vidania* strains from diverse planthoppers based on full-length sequences (1530 shared nucleotide positions) of 16S rRNA gene. Only bootstrap values above 70% are shown. Sequences from the seven Dictyopharidae species studied here are highlighted using the colored font.

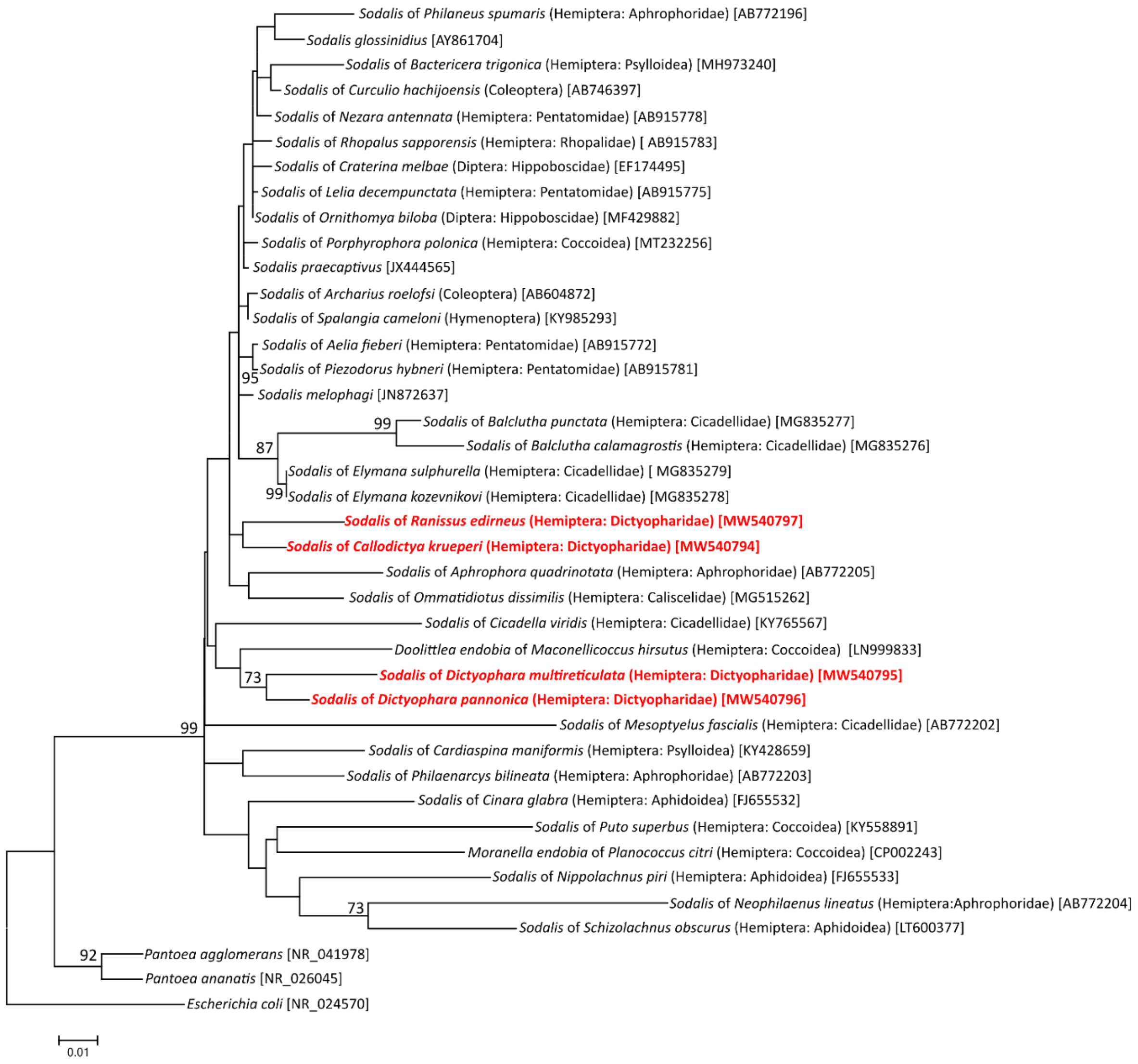

Fig. S1C. ML phylogeny of *Sodalis* strains from different hosts based on 16S rRNA gene sequences. Only bootstrap values above 70% are shown.

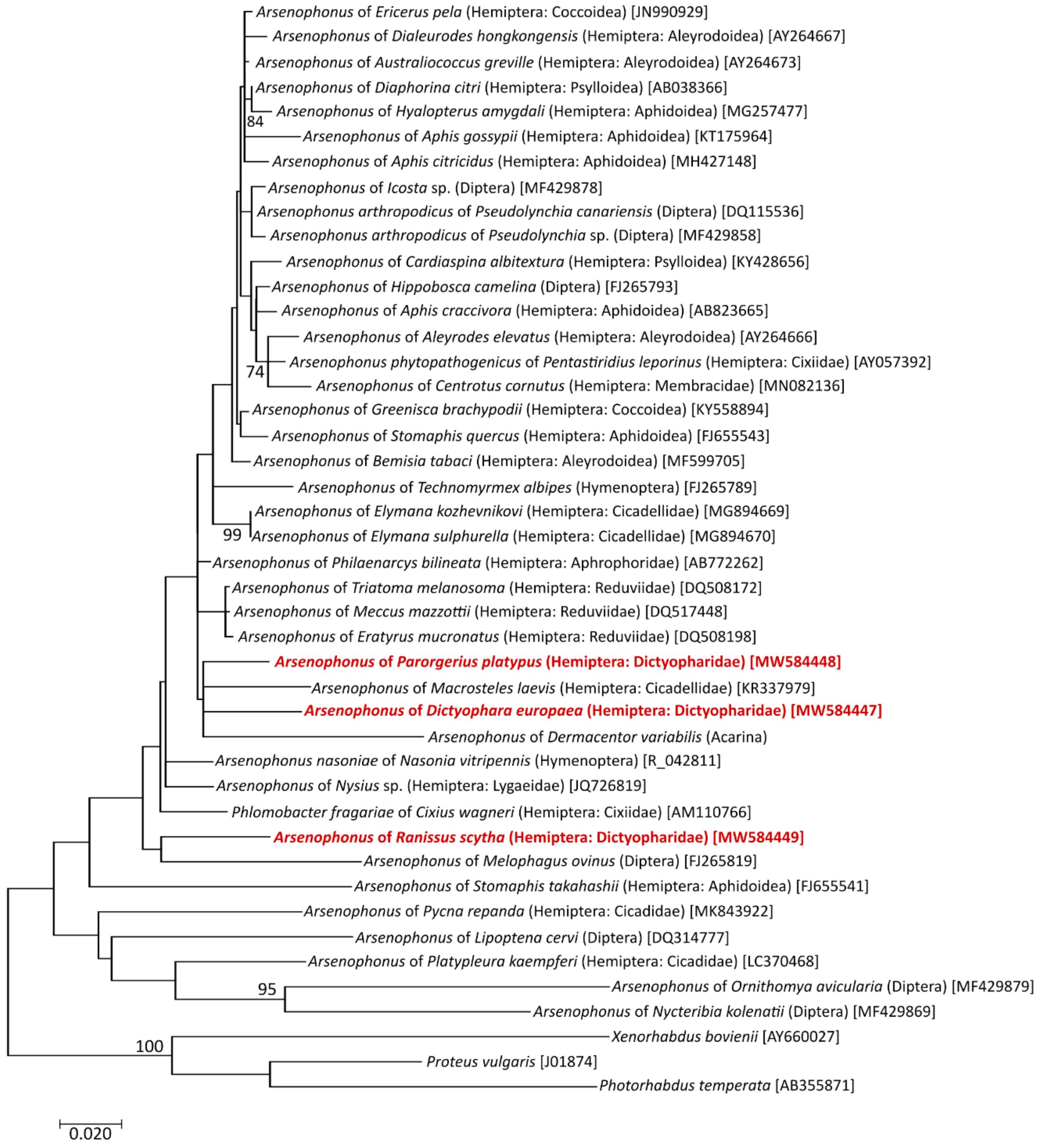

Fig. S1D. ML phylogeny of *Arsenophonus* strains from different hosts based on 16S rRNA gene sequences. Only bootstrap values above 70% are shown.

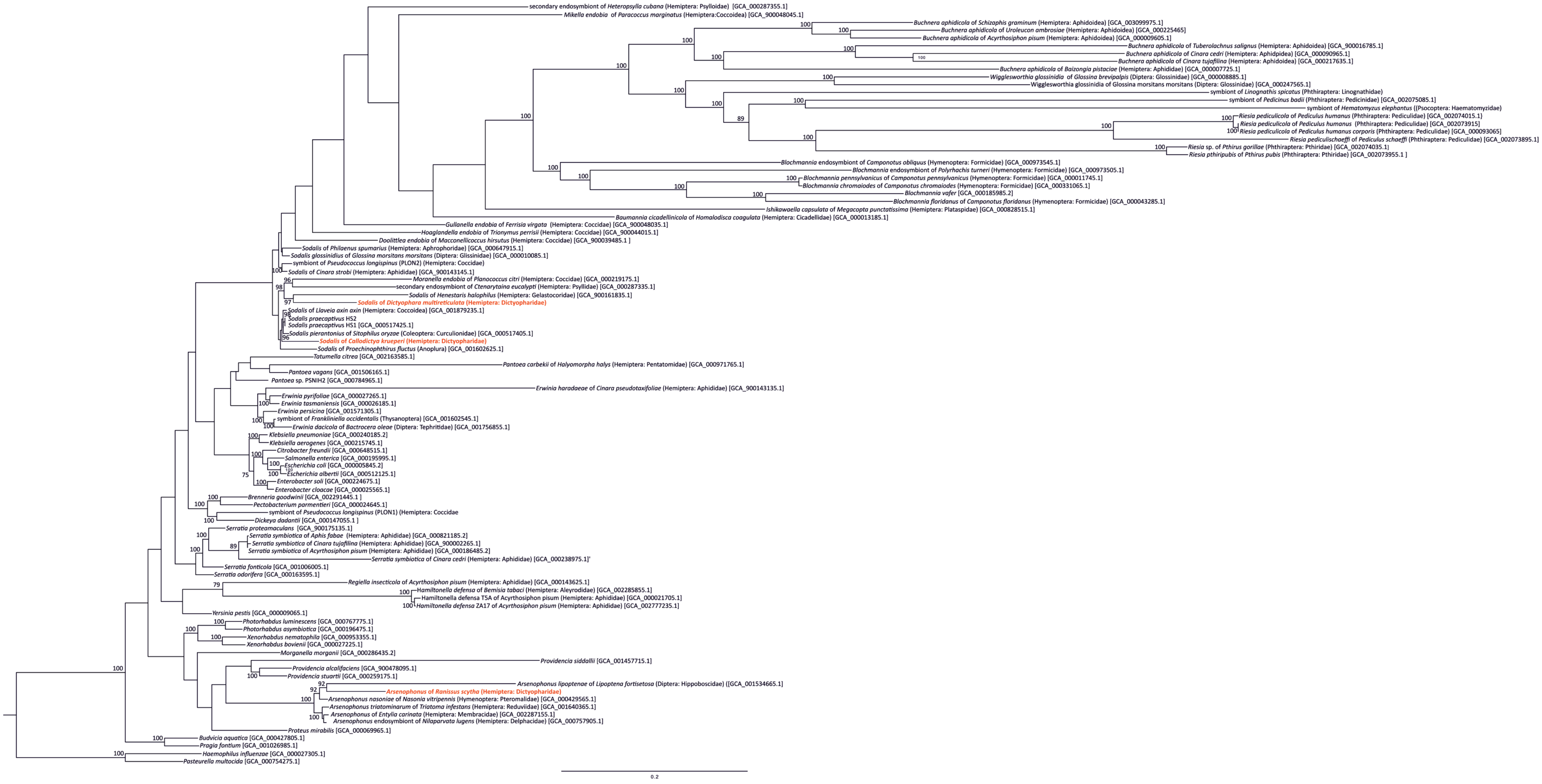

Fig. S1E. ML phylogeny based on 1th and 2th codon positions of 129 conserved single-copy protein-coding genes of symbiotic enterobacteria and relatives. Only bootstrap values above 70% are shown.
