# Supplementary material for: Alternative Transmission Patterns in Independently Acquired Nutritional Cosymbionts of Dictyopharidae Planthoppers
Source: mBio. 2021 Aug 31;12(4):e01228-21. doi: 10.1128/mBio.01228-21 (PMC8406288; doi:10.1128/mBio.01228-21)
Supplement: FIG S3 [file mbio.01228-21-sf003.pdf]

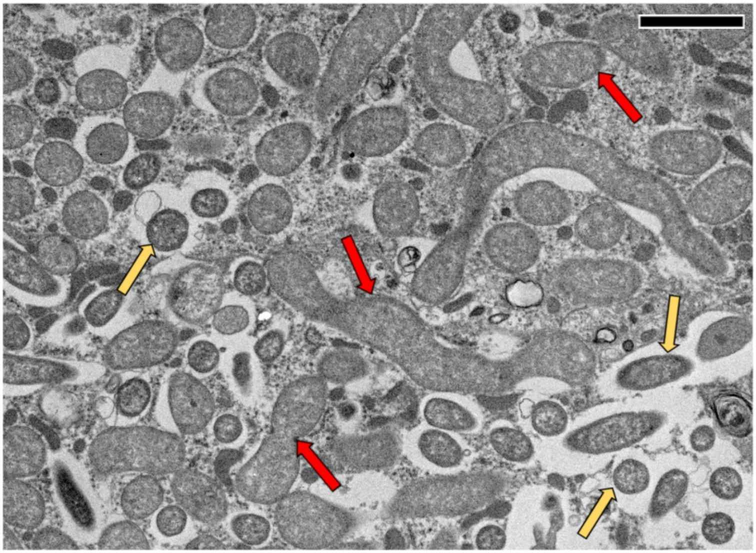

Fig. S3A. *Dictyophara pannonica*. Two morphotypes of *Sodalis* symbiont (red and yellow arrows) within bacteriome of single individuals. TEM, scale bar = 2μm

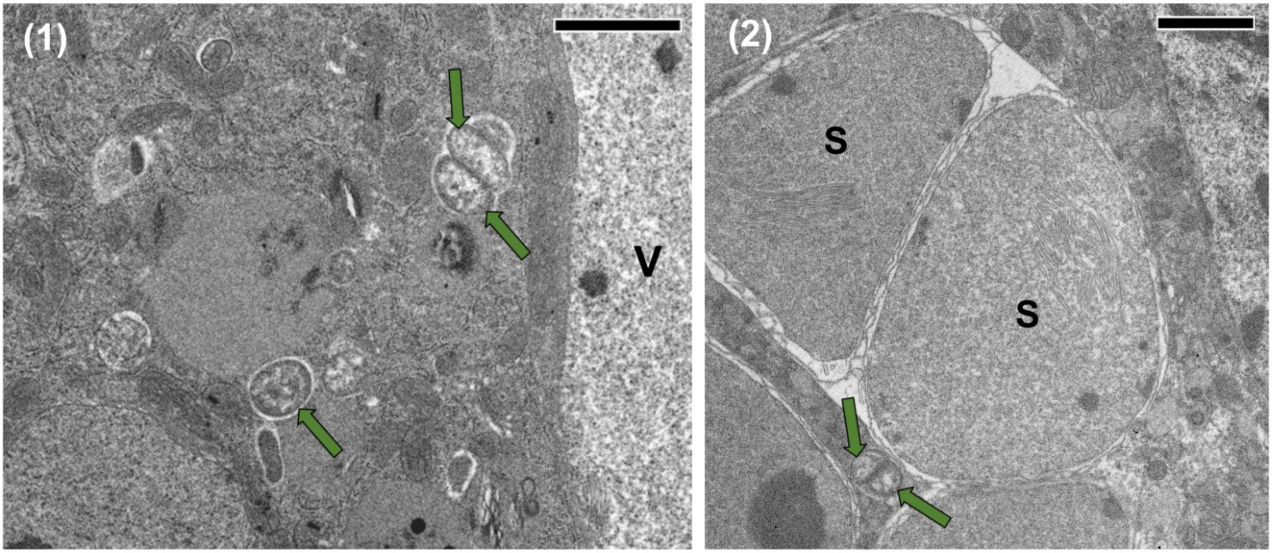

Fig. S3B. Distribution of *Wolbachia* symbiont in the Dictyopharidae species.
Bacteria *Wolbachia* (green arrows) in the cytoplasm of the *Vidania* (1) and *Sulcia* (2) bacteriocytes. (1) *Callodictya krueperi*, (2). *Dictyophara europaea*. TEM, scale bar = 2 μm. S - *Sulcia*, V - *Vidania*
