# Supplementary material for: Alternative Transmission Patterns in Independently Acquired Nutritional Cosymbionts of Dictyopharidae Planthoppers
Source: mBio. 2021 Aug 31;12(4):e01228-21. doi: 10.1128/mBio.01228-21 (PMC8406288; doi:10.1128/mBio.01228-21)
Supplement: FIG S2 [file mbio.01228-21-sf002.pdf]

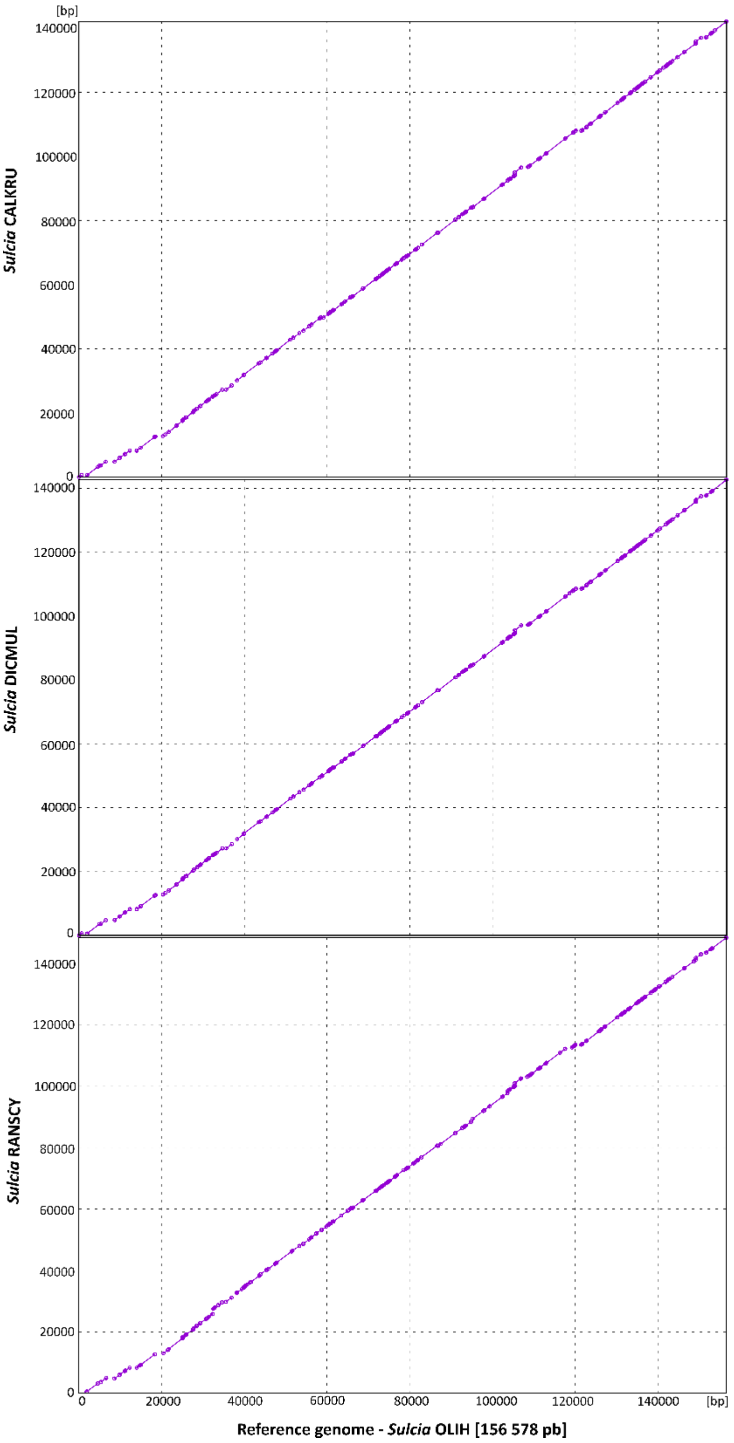

Fig. S2A. PROMER alignments of final *Sulcia* genomes from three Dictyopharidae species: *Callodictya krueperi* (CALKRU), *Dictyophara multireticulata* (DICMUL), and *Ranissus scytha* (RANSCY) against the *Sulcia* genome from *Oliarus filicicola* (OLIH; family Cixiidae).

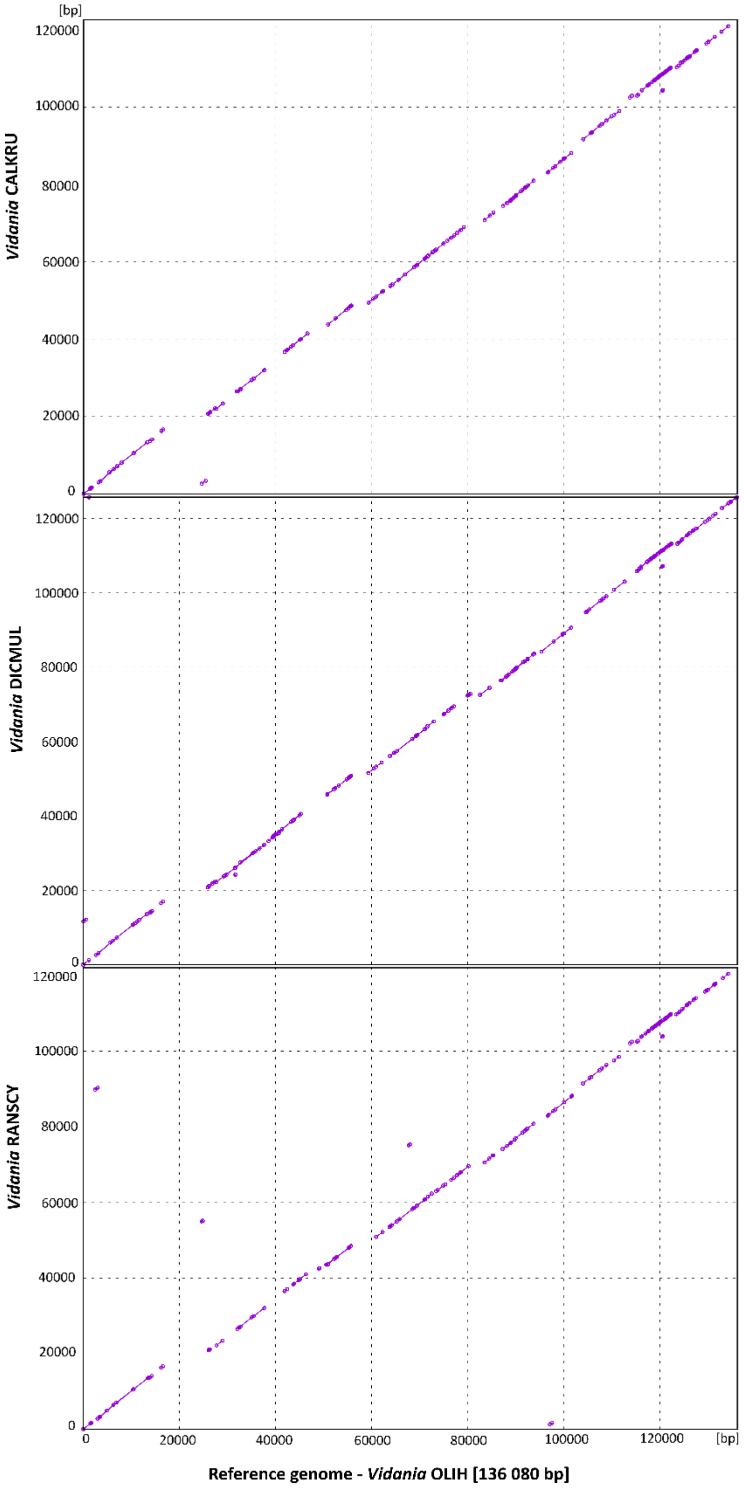

Fig. S2B. PROMER alignments of final *Vidania* genomes from three Dictyopharidae species: *Callodictya krueperi* (CALKRU), *Dictyophara multireticulata* (DICMUL), and *Ranissus scytha* (RANSCY) against the *Vidania* genome from *Oliarus filicicola* (OLIH; Cixiidae).
