# Supplementary material for: Alternative Transmission Patterns in Independently Acquired Nutritional Cosymbionts of Dictyopharidae Planthoppers
Source: mBio. 2021 Aug 31;12(4):e01228-21. doi: 10.1128/mBio.01228-21 (PMC8406288; doi:10.1128/mBio.01228-21)
Supplement: TEXT S1 [file mbio.01228-21-s0001.docx]

Text S1 - Bioinformatic pipelines for the analysis of COI and 16S rRNA amplicon data

**Analysis of COI amplicon data**

COI amplicon data analysis was conducted using mothur v. 1.43.0. The following commands were used.

######## Setting working directories

set.dir(input=/home/anna.michalik/Fulgoromorpha/COI/COI2, output=/home/anna.michalik/Fulgoromorpha/COI/COI2)

######## Creating the file with a list of libraries and files

make.file(inputdir=/home/anna.michalik/Fulgoromorpha/COI/COI2, type=fastq, prefix=DIC_COI)

######## Assembling forward and reverse reads into contigs

make.contigs(file=DIC_COI.files)

######## Quality-trimming sequences

trim.seqs(fasta=DIC_COI.trim.contigs.fasta, oligos=primers_to_trim.oligos, minlength=250, maxlength=500, maxambig=0, maxhomop=10, pdiffs=2)

######## Listing sequences in trimmed fasta file, extracting corresponding sequence IDs from the group file

list.seqs(fasta=DIC_COI.trim.contigs.trim.fasta)

get.seqs(accnos=DIC_COI.trim.contigs.trim.accnos, group=current)

######## Getting basic information about the sequences (e.g., median length, the number of ambiguous bases)

summary.seqs(fasta=current)

######## Checking the number of reads in libraries before and after quality trimming

count.groups(group=DIC_COI.contigs.groups)

count.groups(group=DIC_COI.trim.contigs.fasta)

######## Picking unique sequences

unique.seqs(fasta=DIC_COI.trim.contigs.trim.fasta)

######## Generating a unique sequence table, with info on the abundance of each unique sequence in each library

count.seqs(name=DIC_COI.trim.contigs.trim.names, group=DIC_COI.contigs.pick.groups, compress=f)

######## Discarding singleton sequences

split.abund(fasta=current, count=current, cutoff=1)

######## Getting basic information about the sequences

summary.seqs(fasta=DIC_COI.trim.contigs.trim.unique.abund.fasta)

######## Aligning sequences against a custom database containing reference COI sequences of insects

align.seqs(fasta=DIC_COI.trim.contigs.trim.unique.abund.fasta,reference=COIreference2.fas)

######## Getting the basic information about alignment

summary.seqs(fasta=current)

######## Removing unaligned or poorly aligned sequences

screen.seqs(fasta=DIC_COI.trim.contigs.trim.unique.abund.align, count=DIC_COI.trim.contigs.trim.abund.count_table, minlength=400)

######## Removing any columns containing only gap characters

filter.seqs(fasta=DIC_COI.trim.contigs.trim.unique.abund.good.align,vertical=T, trump=.)

######## Changing file names

rename.file(fasta=current, count=current, prefix=COI)

######## Computing pairwise distance matrix

dist.seqs(fasta=DIC_COI.fasta, cutoff=0.1)

######## Clustering OTUs

cluster(column=current, count=DIC_COI.count_table, cutoff=0.03)

######## Assigning sequences to OTUs based on 97% identity

bin.seqs(list=current, fasta=current, label=0.03)

######## Creating a 97% OTU table

make.shared(list=current, count=current, label=0.03)

**Analysis of 16S rRNA amplicon data**

The analysis of amplicon data for the V4 region of the 16S rRNA gene was conducted using mothur v. 1.43.0. The following commands were used.

######## Setting working directories

set.dir(input=/home/anna.michalik/Fulgoromorpha/DIC2/16Samplicon_analysis, output=/home/anna.michalik/Fulgoromorpha/DIC2/16Samplicon_analysis)

######## Creating the file with a list of libraries and files

make.file(inputdir=/home/anna.michalik/Fulgoromorpha/DIC2/16Samplicon_analysis, type=fastq, prefix=16S)

######## Assembling forward and reverse reads into contigs

make.contigs(file=16S.files)

######## Quality-trimming of sequence data

trim.seqs(fasta=16S.trim.contigs.fasta, oligos=primers_to_trim.oligos, minlength=250, maxlength=500, maxambig=0, maxhomop=10, pdiffs=2)

######## Listing sequences in trimmed fasta file, extracting corresponding sequence IDs from the group file

list.seqs(fasta=16S.trim.contigs.trim.fasta)

get.seqs(accnos=16S.trim.contigs.trim.accnos, group=current)

######## Getting the basic information about the sequences summary.seqs(fasta=current)

######## Checking the numbers of reads in libraries before and after quality trimming

count.groups(group=16S.contigs.groups)

count.groups(group=16S.trim.contigs.fasta)

######## Identifying unique sequences

unique.seqs(fasta=16S.trim.contigs.trim.fasta)

######## Generating a unique sequence table, with info on the abundance of each unique sequence in each library

count.seqs(name=16S.trim.contigs.trim.names, group=16S.contigs.pick.groups, compress=f)

######## Discarding singleton sequences

split.abund(fasta=current, count=current, cutoff=1)

######## Getting basic information about sequences

summary.seqs(fasta=16S.trim.contigs.trim.unique.abund.fasta)

######## Aligning sequences against a SILVA database

align.seqs(fasta=16S.trim.contigs.trim.unique.abund.fasta, reference=/mnt/matrix/symbio/db/silva.nr_v132.align, processors=20)

# * align.seqs(fasta=16S.trim.contigs.trim.unique.abund.fasta, reference=/home/anna.michalik/Fulgoromorpha/DIC2/16Samplicon_analysis/new_internal_reference.fas, processors=20)

# * Note: We processed 16S rDNA amplicon sequencing data, using the command above and the commands below, twice. In the first pass, we aligned unique sequences against Silva v. 132 database and proceeded with that alignment. However, because of the low sequence similarity between some of the planthopper symbiont sequences and the references, the suboptimal alignment resulted in some spurious OTUs. Hence, we chose the representative sequences for all OTUs, aligned them against each other, manually curated the alignment, and used it as the alignment reference during the second pass.

######## Getting basic information about the alignment

summary.seqs(fasta=16S.trim.contigs.trim.unique.abund.align)

######## Removing unaligned sequences

screen.seqs(fasta=16S.trim.contigs.trim.unique.abund.align, count=16S.trim.contigs.trim.abund.count_table, start=1, end=273, minlength=220)

######## Removing any columns containing only gap characters

filter.seqs(fasta=current, vertical=T, trump=.)

######## Re-selecting unique sequences

unique.seqs(fasta=16S.trim.contigs.trim.unique.abund.good.filter.fasta, count=16S.trim.contigs.trim.abund.good.count_table)

######## Chimera filtering using UCHIME

chimera.uchime(fasta=16S.trim.contigs.trim.unique.abund.good.filter.unique.fasta, reference=self, count=16S.trim.contigs.trim.unique.abund.good.filter.count_table, dereplicate=f, mindiv=0.35, processors=20, minh=0.5, xn=3)

######## Removing chimeric sequences

remove.seqs(accnos=current, fasta=current, count=current)

######## Taxonomic classification of sequences

classify.seqs(fasta=current, count=current, reference=/mnt/matrix/symbio/db/silva.nr_v132.align, taxonomy=/mnt/matrix/symbio/db/silva.nr_v132.tax, cutoff=80)

######## Removing sequences not classified as Bacteria

remove.lineage(fasta=current, count=current, taxonomy=current, taxon=Chloroplast-Mitochondria-Archaea-Eukaryota)

######## Getting sequence classification summary

summary.tax(taxonomy=current, count=current)

######## Changing file names

rename.file(fasta=current, count=current, taxonomy=current, prefix=16S)

######## Computing pairwise distance matrix

dist.seqs(fasta=16S.fasta, processors=24, cutoff=0.05)

######## Clustering OTUs using nearest neighbor method

cluster(column=current, count=16S.count_table, cutoff=0.03, method=nearest)

######## Assigning sequences to OTUs based on 97% identity

bin.seqs(list=current, fasta=current, label=0.03)

######## Creating a 97% OTU table

make.shared(list=current, count=current, label=0.03)
